# Accessing Chemo- and Regioselective Benzylic and Aromatic Oxidations by Protein Engineering of an Unspecific Peroxygenase

**DOI:** 10.1021/acscatal.1c00847

**Published:** 2021-06-07

**Authors:** Anja Knorrscheidt, Jordi Soler, Nicole Hünecke, Pascal Püllmann, Marc Garcia-Borràs, Martin J. Weissenborn

**Affiliations:** † Bioorganic Chemistry, Leibniz Institute of Plant Biochemistry, Weinberg 3, 06120 Halle, Germany; ‡ Institute of Chemistry, Martin Luther University Halle-Wittenberg, Kurt-Mothes-Str. 2, 06120 Halle, Germany; § Institut de Química Computacional i Catàlisi and Departament de Química, Universitat de Girona, Carrer Maria Aurèlia Capmany 69, 17003 Girona, Catalonia, Spain

**Keywords:** chemoselectivity, unspecific peroxygenase, protein engineering, naphthoquinone, biocatalysis

## Abstract

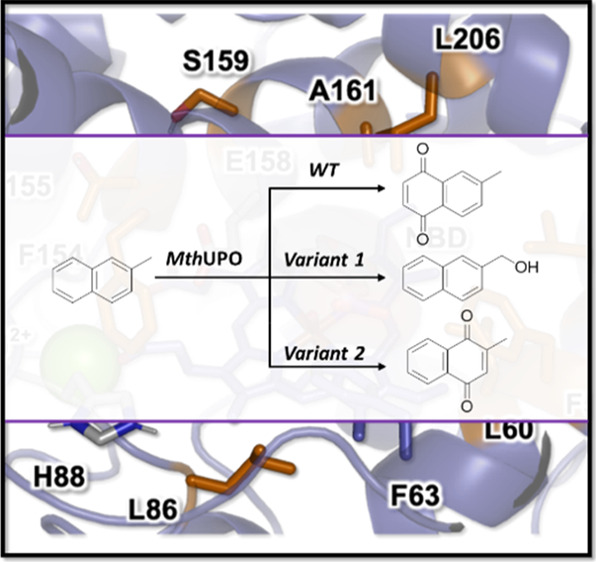

Unspecific peroxygenases (UPOs) enable oxyfunctionalizations of a broad substrate range with unparalleled activities. Tailoring these enzymes for chemo- and regioselective transformations represents a grand challenge due to the difficulties in their heterologous productions. Herein, we performed protein engineering in *Saccharomyces cerevisiae* using the *Mth*UPO from *Myceliophthora thermophila*. More than 5300 transformants were screened. This protein engineering led to a significant reshaping of the active site as elucidated by computational modelling. The reshaping was responsible for the increased oxyfunctionalization activity, with improved *k*_cat_/*K*_m_ values of up to 16.5-fold for the model substrate 5-nitro-1,3-benzodioxole. Moreover, variants were identified with high chemo- and regioselectivities in the oxyfunctionalization of aromatic and benzylic carbons, respectively. The benzylic hydroxylation was demonstrated to perform with enantioselectivities of up to 95% *ee*. The proposed evolutionary protocol and rationalization of the enhanced activities and selectivities acquired by *Mth*UPO variants represent a step forward toward the use and implementation of UPOs in biocatalytic synthetic pathways of industrial interest.

## Introduction

**Scheme 1:**
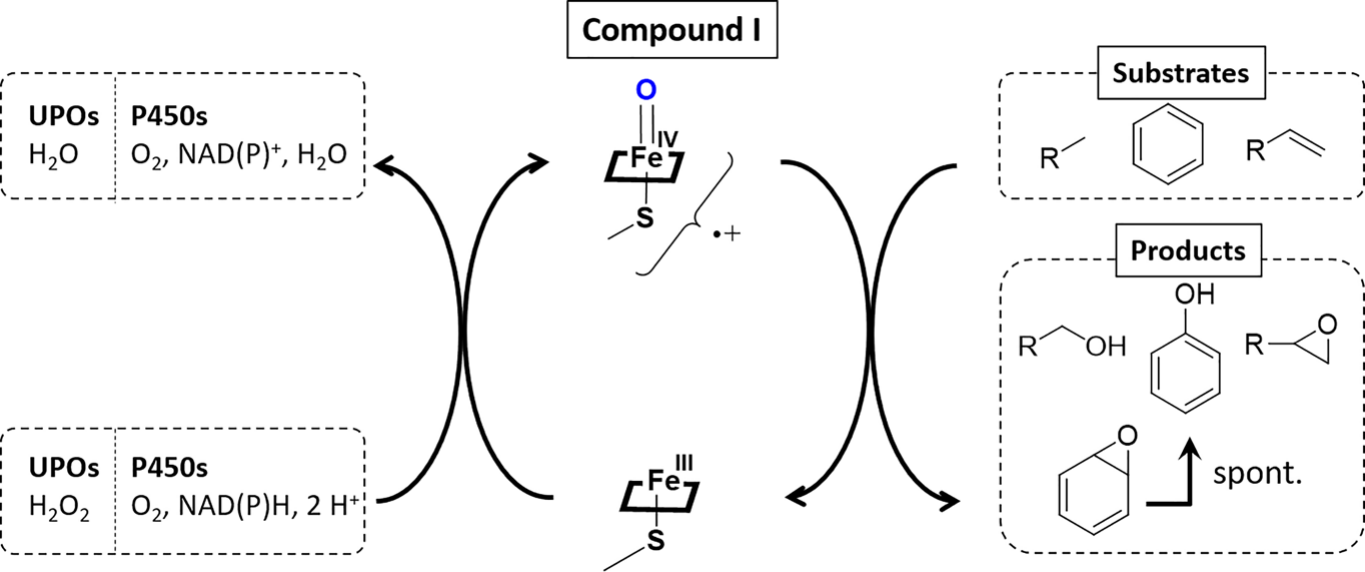
Formation of the Catalytically Active Oxyferryl Species (Cpd I) in P450s and UPOs and the Oxyfunctionalization of sp^2^- and sp^3^-Hybridized Carbons

Fungal unspecific peroxygenases (UPOs) are heme-containing proteins that catalyze oxyfunctionalization reactions of a broad substrate scope via an oxyferryl active species known as compound I (Cpd I), analogous to hemeperoxidases and P450 monooxygenases (P450s).^1−4^ UPOs utilize hydrogen peroxide as a “prereduced” oxygen source and do not require additional reducing agents or reductase domains such as P450s, which require NAD(P)H equivalents and electron transfer steps to activate molecular oxygen (Scheme 1).^5−7^ This facile Cpd I generation and its high activities render UPOs as very promising biocatalysts. UPOs have demonstrated in the last two decades to be highly efficient biocatalysts for carbon, sulfur, and nitrogen oxyfunctionalizations.^8−11^ They can activate C–H bonds of sp^3^-hybridized carbons enabling a homolytic cleavage.

The resulting carbon radical rapidly reacts with the iron-bound OH group in a second step to form the hydroxylation product and recover the resting state Fe(III) center (Scheme 1). The functionalization of C–C double bonds results in an epoxide formation, while for aromatic oxidations, the initial epoxidation follows a spontaneous rearomatization resulting in a formal hydroxylation product. Aromatic oxidations could also lead to the respective quinones, such as naphthoquinone from naphthalene, which is assumed to proceed via the 1-naphthol formation followed by a peroxidase-type single electron oxidation.^12,13^

While the activity and stereoselectivity of UPO-catalyzed reactions are auspicious for future synthetic and industrial applications, low regio- and chemoselectivities mostly afford product mixtures hampering their direct utilization.

Examples for poor regioselectivities by most UPOs are the hydroxylations of saturated fatty acids,^14^ alkanes,^15,16^ steroids,^17^ and vitamin D3.^18^

Low chemoselectivities are observed for unsaturated fatty acids^14^ and a range of linear and cyclic alkenes.^19,20^ Although advances have been made by engineering new P450 variants,^21,22^ there is a need to develop more selective UPOs to overcome these limitations.

The shortcomings of UPOs are mostly addressed by smart substrate selections. Although, for example, toluene leads to a mixture of *ortho*, *para*, and benzylic hydroxylations with *Aae*UPO from *Agrocybe aegerita* (syn. *Cyclocybe aegerita*), utilizing phenylethane resulted in the specific hydroxylation of the benzylic carbon.^19,23^ This change in the selectivity trend was likely due to the higher reactivity of the secondary C(sp^3^)–H of the benzylic phenylethane [estimated bond dissociation energy (BDE) 83.0 kcal/mol compared to the respective primary C–H bond in toluene (BDE 86.7 kcal/mol, Figure S17)].

To increase the applicability of UPOs as useful biocatalysts, accessing substrate-independent and selective hydroxylations is of utmost importance. This selectivity increase could be achieved by protein engineering. Protein engineering encompasses the random or rational variation of enzyme amino acid sequences to alter their properties such as activity, substrate scope, selectivity, stability and tolerance to different reaction conditions. In most cases, altering the protein for desired activities requires the assessment of large enzyme libraries.^2,22,24,25^ The development of a high-throughput compatible, heterologous UPO expression system in *Saccharomyces cerevisiae* enabled protein engineering of UPOs.^26,27^ With this advancement, various impressive directed evolution endeavors were pursued aiming toward improved UPO expression,^27^ neutral drift,^28^ and efficient hydroxypropranolol formation.^29,30^

**Scheme 2:**
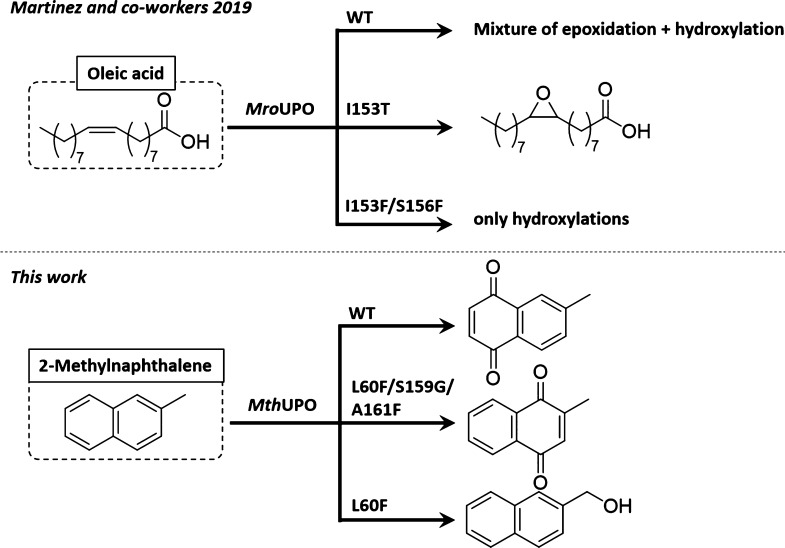
Protein Engineering of UPOs for Chemoselective Oxyfunctionalizations

Recently, a first approach addressing the chemoselectivity issues of UPOs has been reported.^31^ An *Escherichia coli* expression system was developed, which, although thus far not high-throughput capable, allowed first studies on a few point mutations of *Mro*UPO from *Marasmius rotula*. Using molecular dynamics (MD) simulations to explore substrate-binding pathways, heme channel modifications were predicted to influence the epoxidation and hydroxylation, respectively, on the unsaturated fatty acid oleic acid. While the wild type showed hydroxylated and epoxidized products, variant I153T had a strongly enriched epoxide formation. The double mutant I153F/S156F, on the other hand, completely abolished the epoxide formation and exclusively showed hydroxylation regioisomers (Scheme 2).

We have recently established the heterologous expression of a set of UPOs in *S. cerevisiae* and *Pichia pastoris*.^32^ The therein discovered *Mth*UPO from the thermophilic fungus *Myceliophthora thermophila* demonstrated the thus far highest shake flask expression yields allowing a facile microtiter plate-based analysis. Contrary to the well-established *Aae*UPO, *Mth*UPO showed an altered substrate specificity. *Aae*UPO exhibited mostly single hydroxylation of naphthalene to naphthol^33^ and the same applies for the secretion variant *Aae*UPO*.^12,32^*Aae*UPO* also revealed the overoxidation to 1,4-naphthoquinone in small amounts caused by a sequential reaction after the 1-naphthol formation. However, different product ratios are observed for *Mth*UPO. This enzyme catalyzed the naphthalene oxyfunctionalization yielding 1-naphthol and 1,4-naphthoquinone almost in a 1:1 ratio.^32^ For benzylic hydroxylations, *Aae*UPO demonstrated the highest product yields for phenylethane and -propane but strongly abolished and diminished benzylic product formations for phenylbutane and -pentane, respectively. *Mth*UPO showed the opposite tendency: the highest activities were observed for phenylbutane and -pentane and the lowest for phenylethane. Due to the ability of the *Mth*UPO to perform aromatic as well as benzylic hydroxylations efficiently, we hypothesized that by protein engineering, variants could be designed possessing distinct active site geometries enabling the control of the chemoselectivities toward either of the competing transformations. We were interested in 2-methylnaphthalene as a target structure as naphthalene is readily oxidized by *Mth*UPO and therefore provides a good starting point and also has an additional methyl group that offers a position for benzylic hydroxylation. Moreover, the oxidation product 2-methyl-1,4-naphthoquinone is vitamin K_3_ and hence of industrial interest.^34^ To enable a colorimetric high-throughput screening, the 5-nitro-1,3-benzodioxole (NBD) assay^35^ was selected as it utilizes a substrate that bears an aromatic ring system, an sp^3^-carbon, and has a comparable size as naphthalene. A naphthalene-based colorimetric assay would have been based solely on the chemically more facile aromatic hydroxylation and is hence less suitable to identify variants for benzylic and aromatic hydroxylations.

In the present work, a prescreening of single and double saturation libraries was employed to identify relevant positions for activity and selectivity in the active site and the entrance channel of *Mth*UPO. The best-performing variants were selected and combined in a large recombination library. In total, more than 5300 transformants were assessed, which led to the discovery of variants with improved activities. Computational modeling based on extensive MD simulations suggested essential changes in the active site due to mutations that directly impacted preferential substrate binding poses. When naphthalene and its derivatives were tested with the newly engineered variants, different chemoselective oxidation patterns at the benzylic and aromatic positions, respectively, were found. MD simulations described that different catalytically relevant binding poses are explored in each variant by 2-methylnaphthalene, indane, and related tested substrates, which are equivalent to those characterized for the NBD model substrate. The control achieved on the accessible binding poses for the substrates and their specific positioning toward the catalytic Cpd I active species is proposed to be responsible for controlling the chemo- and stereoselectivity observed in these aromatic and benzylic oxidations.

## Results and Discussion

**Figure 1:**
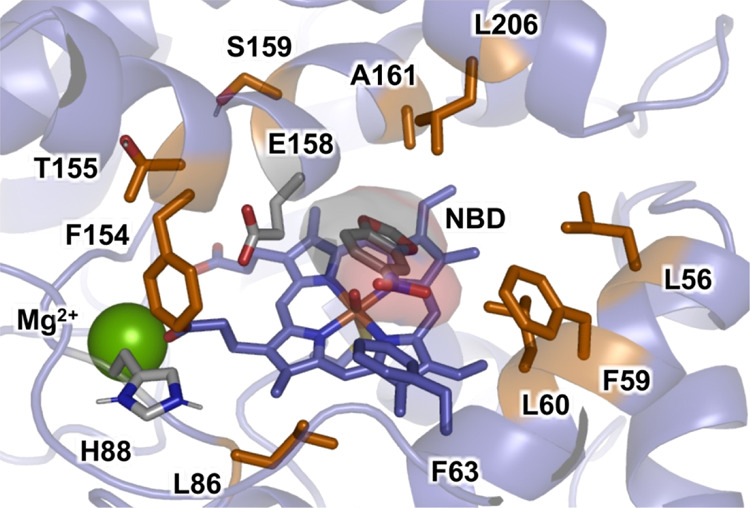
Active site arrangement of *Mth*UPO with the NBD substrate bound obtained from MD simulations based on the generated homology model.^46^ Important active site residues, catalytic residues, and NBD substrate are shown in sticks. The nine positions initially randomized are highlighted in orange, catalytic residues (H88 and E158) and NBD substrate are shown in gray, an important active site residue (F63) is shown in purple, and the structural Mg^2+^ ion is shown as a green sphere.

Based on a homology model for *Mth*UPO, a total of nine positions (L56, F59, L60, L86, F154, T155, S159, A161, and L206) were saturated (Figure 1) using the Golden Mutagenesis technique with the web tool for primer design.^36^ The mutant library was transformed in *S. cerevisiae* producing the corresponding variants. We screened the library using the colorimetric NBD assay^27,35^ in combination with the recently established split-GFP analysis in yeast.^37,48^ The split-GFP^38,48^ system allows the direct quantification of protein concentrations. It consists of a 16 amino acid split-GFP tag, which is C-terminally attached to the protein of interest. By adding a truncated GFP, it recombines with the split-GFP tag and its resulting fluorescence restoration allows the quantification of the split-GFP-carrying protein.

The combination of the NBD and the split-GFP assay enabled the distinction between substrate conversion and protein secretion. Variations at the position L56 substantially influenced the expression of the enzyme. Only 52% of the analyzed transformants displayed a fluorescence response (Table S4). Site saturation at the position L60 yielded improved variants with superior TONs for the NBD conversion. The variants L60M (1.2-fold improvement relative to the wild type), L60Q (1.3-fold), and L60F (2.7-fold) showed the most noticeable improvements (Tables 1 and S5). The variant library of the position F154, which is located at the entrance channel, turned out to be a pivotal position for the NBD conversion.

**Table 1:** Catalytic Activity of *Mth*UPO Variants for the Hydroxylation of NBD^a^

*Mth*UPO variant	conversion [%]	TOF [min^–1^]	TON
WT	29	72	4340
L60F	77	194	11,610
L60F/S159G/A161F	84	210	12,590
F59Q/L60M/S159G/F154A	77	192	11,540
F59Q/L60F/S159G^b^	76	379	22,760

a TOF = turnover frequency, TON = turnover number, standard deviation <3.2%, reaction conditions: 20 nM *Mth*UPO variant, 300 μM NBD, 1 mM H_2_O_2_, 100 mM KPi buffer (pH 7), 5% acetone (v/v), measurement conditions: absorbance was measured at 425 nm for 1 h in triplicates, values were calculated with the corrected extinction coefficient of 10,870 M^–1^ cm^–1^ (see Figure S2).

b 10 nM *Mth*UPO.

Even though 81% of the variants were secreted according to the split-GFP signal, only the rediscovered wild-type enzymes displayed activity (Figure S1, Table S4).

As the second step, we grouped two amino acid residues and saturated them simultaneously with a reduced codon degeneracy (NDT), thereby obtaining the double mutants L60F/F154I and L60F/F154V with a 1.2-fold improvement compared to the wild type (Table S5).

The initial screening of the single and double saturation library provided us with the necessary insights for important residues for enzymatic catalysis. Residues, which had a positive or neutral influence on the NBD conversion, were selected for recombination. These positions and amino acids were F59Q, L60F/Q/M, A57I, F154I/V, S159N/G, and A161I/F. Inspired by Reetz’ single-, double-, and triple-code saturation mutagenesis approaches,^39,40^ we chose to recombine all residues and variations in all combinatorial possibilities including the respective wild-type amino acid.

This combination led to 864 unique variants and required the screening of more than 2300 transformants.

The recombination library resulted in the discovery of triple and quadruple mutations with up to 16.5-fold improved catalytic efficiencies (*k*_cat_/*K*_m_, Table 2). All of the most active variants harbored amino acid exchanges at the L60 position (L60F/M) and the mutation S159G. The kinetic measurements revealed 8.2-fold (F59Q/L60F/S159G), 10.8-fold (F59Q/L60M/S159G/F154A), and 16.5-fold (L60F/S159G/A161F) increased *k*_cat_/*K*_m_ values relative to the wild type (Table 2). Although the *K*_m_ value of NBD was decreased or similar to the wild type, the values were significantly increased for H_2_O_2_ (Table S6). The *k*_cat_ value for NBD, however, was substantially improved for the identified triple and quadruple mutants with an 18.6-fold increase for the variant L60F/S159G/A161F relative to the wild type.

**Table 2:** Biochemical Characterization of the *Mth*UPO Wild Type and the Evolved Variants toward the Substrate NBD^a^

*Mth*UPO variant	*K*_m_ [μM]	*k*_cat_ [s^–1^]	*k*_cat_/*K*_m_ [M^–1^ s^–1^]
WT	386	7.1	1.9 × 10^4^
L60F	110	2.9	2.7 × 10^4^
L60F/S159G/A161F	422	132.2	3.1 × 10^5^
F59Q/L60M/S159G/F154A	290	59.4	2.1 × 10^5^
F59Q/L60F/S159G	303	47.0	1.6 × 10^5^

a Standard deviation for the kinetics <18%, values were calculated with the corrected extinction coefficient of 10,870 M^–1^ cm^–1^. For reaction conditions, see the Materials and Methods. For Michaelis–Menten plots, see the Supporting Information.

**Figure 2:**
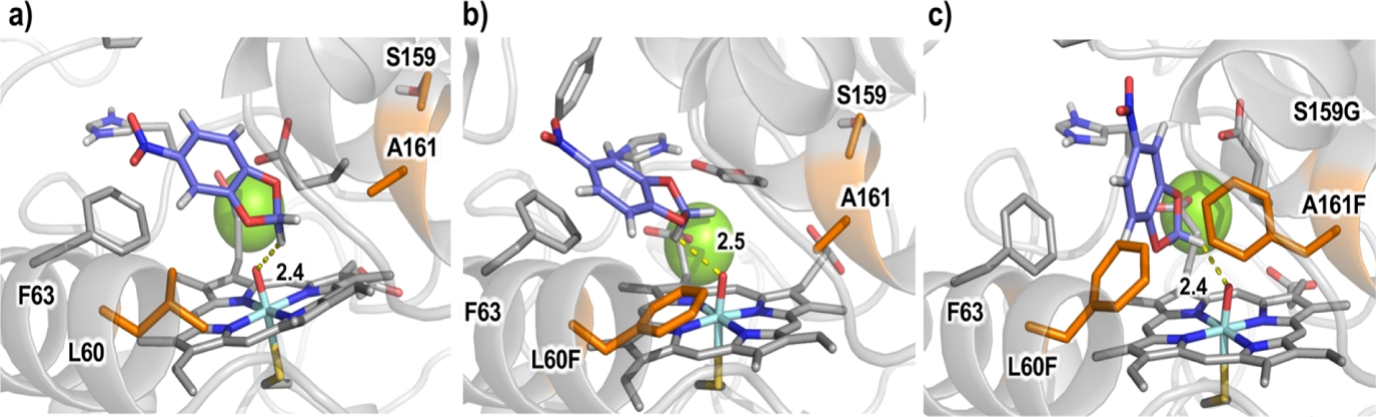
Active site arrangement of *Mth*UPO and evolution of NBD’s catalytically relevant binding modes in (a) wild type and the variants (b) L60F and (c) L60F/S159G/A161F as observed from MD simulations. Substrate, heme cofactor, and important active site and catalytic residues are shown in sticks. The three mutated positions are highlighted in orange, the NBD substrate is shown in purple, and the structural Mg^2+^ ion is shown as a green sphere.

At this stage, we were interested in gaining some understanding of the changes induced by the mutations. Starting from the homology model for the *Mth*UPO structure based on the solved crystal structure of the *Mro*UPO (PDB: 5FUJ, 33.6% identity), we refined it by extensive MD simulations accumulating a total of 5 μs (5000 ns) of simulation time (see the Materials and Methods for details). We then performed docking calculations that were used to obtain starting points for substrate-bound MD simulations in order to analyze the binding of NBD in the wild type and the selected variants L60F and L60F/S159G/A161F (Figures 2, S9 and S10). These simulations revealed a switch in the binding mode of NBD when moving from wild type to L60F and L60F/S159G/A161F. Due to the inclusion of bulkier, aromatic residues in the inner active site—first L60F and then A161F—the NBD substrate was reoriented from its original more buried binding pose in the wild type to partially occupying the entrance channel in L60F/S159G/A161F. This new binding pose allows the NBD to better approach the oxyferryl Cpd I species in a near attack conformation for C–H abstraction, thus facilitating the oxidation reaction.

**Scheme 3:**
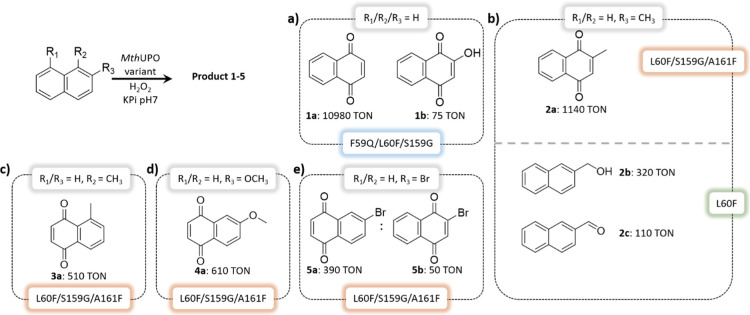
Catalytic Activity of *Mth*UPO Variants for the Hydroxylation of Naphthalene and Its Derivatives; (a) Transformation of Naphthalene with the Variant F59Q/L60F/S159G to 1,4-Naphthoquinone **1a** and under Altered Conditions to Lawsone (**1b**), (b) 2-Methylnaphthalene Oxyfunctionalization with the Variant L60F/S159G/A161F Predominantly to 2-Methyl-1,4-naphthoquinone (**2a**, vitamin K_3_) and Using the Variant L60F towards the Benzylic Functionalization Leading to **2b** and **2c**; (c–e) Transformation with the Variant L60F/S159G/A161F of 1-Methyl-, 2-Methoxy-, and 2-Bromonaphthalene, Respectively, Led to the Formation of the Naphthoquinone Derivatives **3a**, **4a**, and **5a**; Only 2-Bromonaphthalene also Revealed Significant Oxyfunctionalizations of the Substituted Ring to Form **5b** Reaction conditions were 1 mM substrate, 4/5 mM H_2_O_2_ (end concentration), 50–500 nM *Mth*UPO variant, 100 mM KPi buffer pH 7, 5% (v/v) acetone. Addition of 100–200 μL of an 8–16 mM H_2_O_2_ stock solution via a syringe pump within 1–2 h and additional stirring for 30 min to overnight. Details are shown in Table S7. Standard deviation of triplicates <5.2%.

With these new engineered variants possessing different active site cavities, we were intrigued to probe their influence on the regio- and chemoselectivity on naphthalene derivatives (Scheme 3). The most active variants characterized from the NBD colorimetric assay were selected, and an initial screening on a substrate panel based on naphthalene and its derivatives was performed by gas chromatography–mass spectrometry (GC–MS), resulting in significant changes in the oxidation patterns (Figures S4–S6).

Utilizing a syringe pump setup achieved more than 10,000 TONs with variant F59Q/L60F/S159G for the conversion of naphthalene to 1,4-naphthoquinone **1a** (Scheme 3a). By increasing H_2_O_2_ equivalents, 2-hydroxy-1,4-naphthoquinone **1b** was formed as a byproduct, which is a natural dye known as Lawsone.^41^ Naphthalene conversion by the yeast expression variant of *Aae*UPO was previously shown to result in the predominant formation of 1- and 2-naphthol and 1,4-naphthoquinone only as a minor byproduct.^12^

The biotransformation of 2-methylnaphthalene with the wild type led predominantly to 6-methyl-1,4-naphthoquinone (Figure S7). The variant L60F/S159G/A161F was able to shift the major product formation to 2-methyl-1,4-naphthoquinone (**2a**), also known as vitamin K_3_, demonstrating the regioselectivity of this variant (Scheme 3b). We were pleased that we also identify the L60F variant with the preference for the methyl hydroxylation of 2-methylnaphthalene. This variant showed altered chemoselectivity dramatically suppressing the aromatic hydroxylation of the naphthalene core while accessing the hydroxylation of the methyl group (**2b**) as the major product. Also, the overoxidation to the aldehyde was observed (**2c**, Scheme 3b).

**Figure 3:**
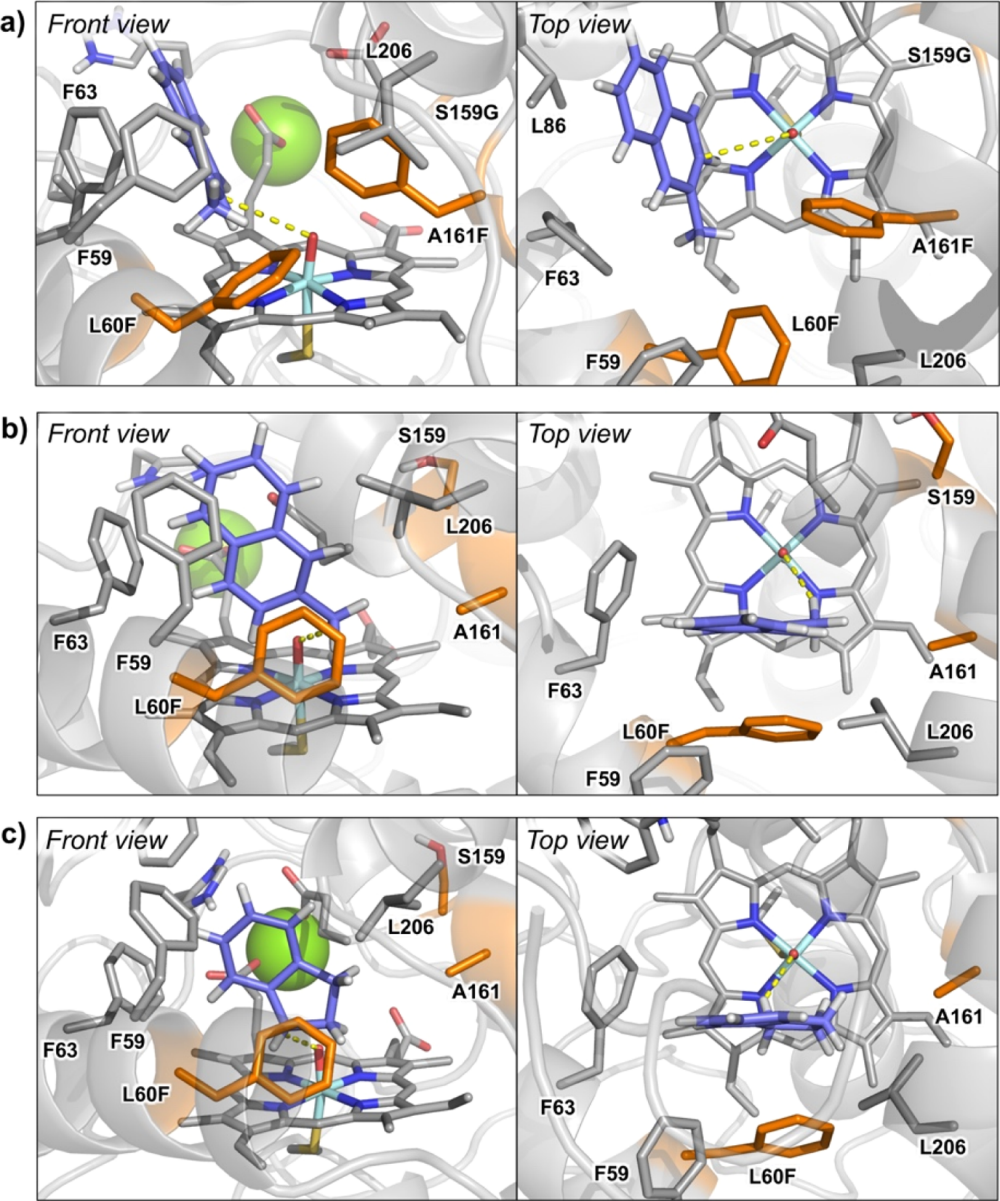
Catalytically relevant binding modes as characterized from MD simulations of (a) 2-methylnaphthalene in the L60F/S159G/A161F variant; (b) 2-methylnaphthalene in the L60F variant; and (c) indane in the L60F variant. Substrates, heme cofactor, and important active site and catalytic residues are shown in sticks. The three mutated positions are highlighted in orange, substrates are shown in purple, and the structural Mg^2+^ ion is shown in a green sphere.

To understand the switch in chemoselectivity when moving from L60F to L60F/S159G/A161F variants, we performed docking and MD simulations with 2-methylnaphthalene bound in both enzymes. MD simulations demonstrated that when 2-methylnaphthalene is bound in the L60F variant, only the methyl group was able to approach the active Fe=O species in a catalytically competent pose due to the presence of the bulky phenylalanine residue L60F (Figures 3 and S11). On the other hand, when 2-methylnaphthalene was bound into the variant L60F/S159G/A161F, a binding pose similar to the previously observed NBD positioning in this variant was observed (Figures 3 and S12). This change in substrate positioning is promoted by hydrophobic interactions occurring in the newly engineered active site, which is dominated by the presence of several aromatic residues (F59, F60, F63, and F161). Within this new binding pose, the substituted aromatic ring of 2-methylnaphthalene is placed close enough to the oxyferryl (Cpd I) catalytic species to react while keeping the methyl group away from it. The different binding modes preferentially explored by 2-methylnaphthalene in these two variants are responsible for the observed switch in chemoselectivity (Figures S12 and S13).

When 1-methylnaphthalene was considered, a decrease in the TONs of 50% was observed, yielding the oxidation of the unsubstituted ring (Scheme 3c), as opposite to 2-methylnaphthalene. More surprisingly, also, substitutions at the 2 position of the naphthalene core led to the oxidation of the unsubstituted ring generating the 6-methoxy- (**4a**, Scheme 3d) and 6-bromo-1,4-naphthoquinone (**5a**, Scheme 3e) products with diminished TONs relative to 2-methylnaphthalene. For 2-bromo-naphthalene, 2-bromo-1,4-naphthoquinone (**5b**) was also detected as a byproduct.

To rationalize the different oxidation patterns observed for 1- and 2-substituted naphthalene derivatives, MD simulations were carried out. Simulations with the variant L60F/S159G/A161F and 2-methoxy-naphthalene (Figures S13 and S14) revealed a preferential binding of the substrate in a region of the active site equivalent to the one observed for 2-methylnaphthalene. However, due to the presence of the bulkier methoxy group at the 2 position and the presence of the L60F residue, the naphthalene core in 2-methoxy-naphthalene needs to rotate slightly when approaching the Fe=O active species in a catalytically competent pose. This rotation places the 2-methoxy group away from the L60F and the heme cofactor and brings the unsubstituted aromatic ring closer to the Fe=O active species. Due to this proximity, a switch in the regioselectivity as compared to 2-methylnaphthalene occurred. A similar behavior was also observed for 1-methylnaphthalene when bound in the variant L60F/S159G/A161F in a catalytically competent pose (Figure S15).

Intrigued by the chemoselective benzylic hydroxylation catalyzed by L60F, we tested this variant for the benzylic oxidation of indane and tetralin (1,2,3,4-tetrahydronaphthalene) investigating additional stereoselective control. L60F was able to convert indane with more than 8000 TONs and improved enantioselectivity for the (*R*)-1-indanol (**5a**) from 85% ee (wild type) to 95% ee (Tables 3 and S8). Interestingly, the examination of the variants L60F/S159G/A161F and F59Q/L60M/S159G/F154A revealed the loss of enantioselectivity and the excess in the formation of the (*S*)-enantiomer (Table S8). MD simulations with indane bound in the variant L60F characterized a preferential binding pose of indane that resembles the previously observed pose for 2-methylnaphthalene in L60F (Figures 3 and S16). Simulations showed that indane mainly interacts with the aromatic rings of F63 and L60F, establishing C–H···π interactions. These hydrophobic interactions keep the substrate in a preferred binding pose where only the pro-*R* C(1)–H of the indane is close enough and well-aligned to the oxyferryl species to be efficiently hydroxylated.

**Table 3:**
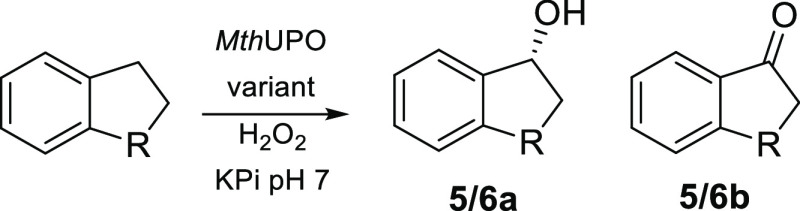

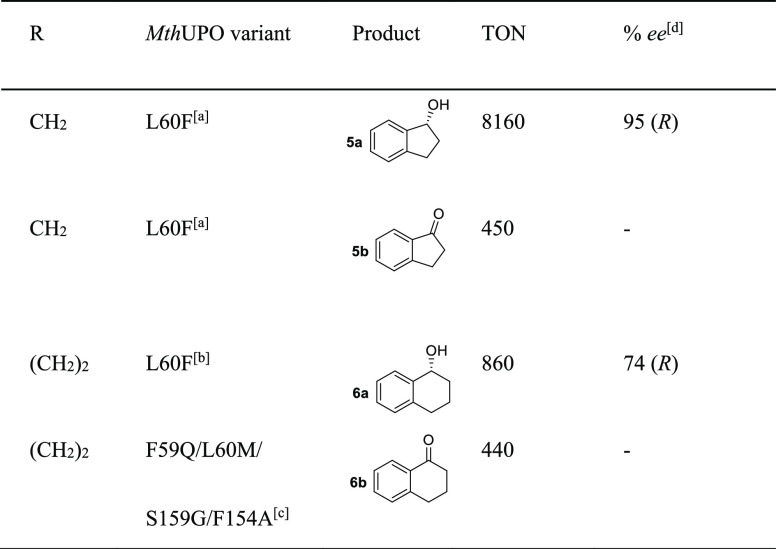
Catalytic Activity of *Mth*UPO Variants toward Benzylic Hydroxylation Yielding Chiral Products^a^

a TON = turnover number, standard deviation <6.5%, reaction conditions: 100 nM *Mth*UPO variant, 1 mM indane, 1 mM H_2_O_2_, 100 mM KPi buffer (pH 7), 5% acetone (v/v), 1 h at 25 °C in triplicates.

b 100 nM *Mth*UPO variant, 1 mM 1,2,3,4-tetrahydronaphthalene, 1 mM H_2_O_2_, 100 mM KPi buffer (pH 7), 5% acetone (v/v), 1 h at 25 °C in triplicates.

c 100 nM *Mth*UPO variant, 1 mM 1,2,3,4-tetrahydronaphthalene, 2 mM H_2_O_2_, 100 mM KPi buffer (pH 7), 5% acetone (v/v), 2 h at 25 °C in triplicates.

d Determined by chiral GC.

For the bioconversion of tetralin to the alcohol **6a**, a substantially improved enantioselectivity for the (*R*)-enantiomer was detected for the variant L60F (74% ee) relative to the wild type (45% ee, Figure S8). Similar to indane, a variant could be identified, which forms the (*S*)-enantiomer (F59Q/L60M/S159G/F154A) predominantly.

## Conclusions

The most important landmarks of UPOs are their discovery,^8^ the development of smart hydrogen peroxide delivery systems^2,19,42^ that allow their implementation in biocatalytic processes using milder reaction conditions, and their heterologous expression making them easily accessible.^26,27,31,32^ The next step toward the broader applicability of UPOs as useful biocatalysts is to access more chemo-, regio-, and stereoselective engineered UPO variants.

In this work, the recently characterized *Mth*UPO was engineered using a protein evolution protocol that involves site saturation mutagenesis and an extensive recombination library, making use of a split-GFP and colorimetric assays and a high-throughput yeast expression system that allowed us to screen more than 5300 transformants. This led to *Mth*UPO variants that achieve regioselective aromatic oxidations as well as chemoselective benzylic hydroxylations with 2-methylnaphthalene and other naphthalene derivatives.

Extensive MD simulations indicated that the origin of selectivity in these engineered *Mth*UPO variants is a reshaping of the active site cavity that controls which specific binding modes are accessible for the tested aromatic substrates.

The newly engineered variant L60F/S159G/A161F binds 2-methylnaphthalene in a catalytically relevant conformation that allows almost exclusively oxidation at the substituted ring. This binding mode yields the overoxidized 2-methyl-1,4-naphthoquinone (vitamin K_3_) product. Vitamin K_3_ (menadione) and the vitamin K family comprise 2-methyl-1,4-naphthoquinone derivatives, and the engineered selectivity provides a one-step direct route to access these molecules.

On the other hand, the variant L60F possesses an active site that does not allow the aromatic moiety of 2-methylnaphthalene to productively approach to the reactive oxyferryl species, preferentially binding the substrate in a pose where only the benzylic position can react. This resulted in a highly chemoselective benzylic hydroxylation and partly overoxidation to the aldehyde. The variant L60F also exhibits improved enantioselective benzylic hydroxylation of indane, which is shown to preferentially bind in this variant in a catalytically competent pose similar to the one observed for 2-methylnaphthalene.

The presented work demonstrates the protein engineering toward chemo-, regio-, and enantioselective oxyfunctionalizations catalyzed by fungal UPOs, paving the way toward the broader application of UPOs in enzyme cascades, organic chemistry, and industry.

## Materials and Methods

### Chemicals

Solvents were used as provided without further purification from Carl Roth (Karlsruhe, DE) as GC ultragrade. The commercially available compounds were also used without further purification from the following suppliers: *N*,*O*-bis(trimethylsilyl)trifluoroacetamide (BSTFA, Macherey-Nagel, Düren, DE), hydrogen peroxide solution [30% (w/w) in H_2_O, Sigma-Aldrich, St. Louis, US], cumene hydroperoxide (contains 20% aromatic hydrocarbon, TCI, Tokyo, JP), *tert*-butyl hydroperoxide (70% in water, TCI, Tokyo, JP), NBD (98%, Sigma-Aldrich, St. Louis, US), naphthalene (99%, Sigma-Aldrich, St. Louis, US), (*R*)-(−)-1-indanol (99%, Sigma-Aldrich, St. Louis, US), (*S*)-(+)-1,2,3,4-tetrahydro-1-naphthol (98%, TCI, Tokyo, JP), 1,2,3,4-tetrahydronaphthalene (99%, Fluka, Buchs, CH), 1-indanone (99%, Sigma-Aldrich, St. Louis, US), indane (95%, TCI, Tokyo, JP), 1-hydroxyindane (99%, TCI, Tokyo, JP), α-tetralone (96%, Fluka, Buchs, CH), 2-methylnaphthalene (97%, Sigma-Aldrich, St. Louis, US), 2-naphthaldehyde (98%, TCI, Tokyo, JP), 2-hydroxymethylnaphthalene (97%, abcr, Karlsruhe, DE), 1-methylnaphthalene (96%, TCI, Tokyo, JP), 2-methyl-1,4-naphthoquinone (98%, Sigma-Aldrich, St. Louis, US), 1,4-naphthoquinone (97%, Sigma-Aldrich, St. Louis, US), 2-methoxy-1,4-naphthoquinone (98%, Sigma-Aldrich, St. Louis, US), 1,2,3,4-tetrahydro-1-naphthol (97%, Sigma-Aldrich, St. Louis, US), 2-bromo-1,4-naphthoquinone (98%, Sigma-Aldrich, St. Louis, US), 4-octanone (98%, TCI, Tokyo, JP), 2-methoxynaphthalene (98%, TCI, Tokyo, JP), and 2-bromonaphthalene (97%, Fluka, Buchs, CH). As a buffer system, 50 mM potassium phosphate (KPi) pH 7 was utilized as an aqueous phase for the bioconversions.

### Gas Chromatography–Mass Spectrometry

Measurements were performed on a Shimadzu GCMS-QP2020 NX instrument (Shimadzu, Kyoto, JP) with a Lipodex E column (25 m × 0.25 mm, Macherey-Nagel, Düren, DE) for the chiral and on an SH-Rxi-5Sil MS (30 m × 0.25 mm, Shimadzu, Kyoto, JP) for the achiral measurements, whereas helium was utilized as the carrier gas. The samples were injected split-less (1 μL) with a liner temperature of 280 °C. The interface temperature was set to 290 °C. Ionization was obtained by electron impact with a voltage of 70 V, and the temperature of the ion source was 250 °C. The oven temperature profile for each compound is shown in Table S2. The detector voltage of the secondary electron multiplier was adjusted in relation to the tuning results with perfluorotributylamine. The GC–MS parameters were controlled with GCMS Real Time Analysis, and for data evaluation, GCMS Postrun Analysis (GCMSsolution Version 4.45, Shimadzu, Kyoto, JP) was used. Calibration and quantification were implemented in the selected ion monitoring (SIM) mode with the corresponding *m*/*z* traces, as shown in Table S2, in triplicates. As an internal standard, 4-octanone (1 mM, *m*/*z* 128) in EtOAc was utilized. The product formation of 5-methyl-1,4-naphthoquinone (**3a**) was confirmed by the consistent literature fragmentation pattern:^43^ MS (EI) *m*/*z* 172, 144, 118, 116, 115, 90. For product quantification of 5-methyl-1,4-naphthoquinone (**3a**), 6-methoxy-1,4-naphthoquinone (**4a**, MS (EI) *m*/*z*: 188, 160, 134, 106, 63), and 6-bromo-1,4-naphthoquinone (**5a**, MS (EI) *m*/*z*: 384, 382, 296, 294, 266, 264, 73), the corresponding structural isomers menadione (**2a**), 2-methoxy-1,4-naphthoquinone (**4b**), and 2-bromo-1,4-naphthoquinone (**5b**) were utilized. Compound **1b** was derivatized according to the standard procedure of Macherey-Nagel (Düren, DE) with BSTFA: therefore, the water was removed using an Eppendorf concentrator 5301 (Hamburg, DE) under vacuum at 60 °C for 1.5 h. The resulting residue was dissolved in 100 μL of pyridine with ultrasound for 5 min, followed by the addition of 100 μL of BSTFA. The derivatization reaction was accomplished at 80 °C for 20 min and the solution was directly utilized for GC–MS analysis resulting in the literature known fragmentation pattern:^44^ MS (EI) *m*/*z* 231, 203.

### Single-Site Saturation Mutagenesis

Mutagenesis was performed using the Golden Mutagenesis technique^36^ combined with the “22c-trick”^45^ for residue randomization. The transformations into *S. cerevisiae* were performed as described before.^32^

### Double-Site Saturation Mutagenesis

The single-site saturation mutagenesis led to the identification of relevant amino acid residues, which were combined in a double-site saturation mutagenesis approach based on spatial proximity and their potential interactions. The positions L60/F154, I52/A57, F59/L150, S159/A161, and F63/L86 were therefore simultaneously randomized using an NDT codon degeneracy utilizing the previously mentioned Golden Mutagenesis protocol.

### Generation of a Recombination Library of the Best-Performing Variants

The best-performing variants from the single- and double-site saturation mutagenesis were chosen for recombination. For this approach, the best-performing amino acid residues were selected and randomized. The following positions were chosen: L60 (WT/F/Q/M), F59 (WT/Q), A57 (WT/I), F154 (WT/V/I), S159 (WT/G/N), and A161 (WT/I/F). The library was generated based on the previously mentioned Golden Mutagenesis protocol yielding 864 possible combinations.

### Microtiter Plate Cultivation of *S. cerevisiae*

The *Mth*UPO production was performed as described previously.^32^ The cell pellet was resuspended in the remaining supernatant, and glycerol was added to achieve a final concentration of 25% (v/v). The sealed microtiter plates were frozen by liquid nitrogen and stored at −80 °C as a mother plate for subsequent hit verification.

### Activity Screening via NBD Assay in the Microtiter Plate Format

The utilization of 5-nitro-1,3-benzodioxole for a colorimetric screening approach has been described before yielding the chromophore 4-nitrocatechol.^27,35^ The conditions were slightly modified: after centrifugation, 20 μL of the supernatant from the microtiter plate cultivation was transferred to a polypropylene 96-well screening plate (Greiner Bio-One, Kremsmünster, AT) and 180 μL of the master mix was added [end concentrations: 100 mM KPi buffer pH 7, 300 μM NBD, 1 mM H_2_O_2_, 5% (v/v) acetone]. The absorption was detected for 1 h at 425 nm (interval: 30 s) starting directly after the addition of the master mix using an absorbance reader (TECAN, Grödig, AT). The reaction endpoint was determined overnight. Improved *Mth*UPO variants were identified by comparing the absorption values of the NBD assay and the fluorescence values of the split-GFP assay with the parental variant.

### Split-GFP Assay

Protein normalization was performed employing the principle of a split-GFP normalization assay as described by Santos-Aberturas et al.^47^ with slight modifications as reported previously.^32^ Based on the split-GFP assay, the percentage of secreted variants could be calculated (Table S4).

### Automated Data Evaluation and Verification

For the microtiter plate screening, including the NBD and split-GFP assay, an automated data evaluation by R Studio was utilized. Thereby, the best-performing variants were identified based on their respective endpoint after 1 h and overnight, their NBD slope, and their NBD/GFP correlation when compared to the parental variant. For the data verification, the best-performing variants were reproduced in a microtiter plate setup in triplicates. If the improved activity was confirmed, the protein was cultivated in the shake flask scale and purified for further characterization.

### Shake Flask Cultivation and Protein Purification

The cultivation was performed as previously described.^32^ The samples were stored at −20 °C until further utilization.

### NBD Assay with a Purified Enzyme for TOF/TON Determination

The purified *Mth*UPO variants were measured via the NBD assay under the following conditions: 20 nM *Mth*UPO variant (exception: F59Q/L60F/S159G with 10 nM), 300 μM NBD, 1 mM H_2_O_2_, 100 mM KPi buffer (pH 7), 5% acetone (v/v). The turnover numbers (TONs), turnover frequency values, and conversions were determined after 1 h. For determination of the catalytic performance, the TON, TOF, and conversion were calculated based on the corrected extinction coefficient of 4-nitrocatechol. The actual extinction coefficient was calculated by a calibration curve of 4-nitrocatechol in 100 mM KPi buffer (pH 7) with 5% acetone (v/v) (Figure S2a) leading to ε_425nm, 4-nitrocatechol_ = 11,289 M^–1^ cm^–1^. This coefficient was corrected by the extinction coefficient of NBD ε_425nm, NBD_ = 419 M^–1^ cm^–1^ (Figure S2b) yielding to the corrected coefficient of ε_425nm, corr_ = 10,870 M^–1^ cm^–1^, which can be utilized for the catalytic performance calculations
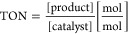

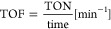


### Determination of Kinetic Parameters

To determine the kinetic parameters *K*_m_ and *k*_cat_, the purified protein samples were utilized. The best-performing variants from the NBD screening were compared to each other: the enzyme concentration was set to 20 nM for each variant for the *K*_m_ (NBD) determination. The only exception was the variant L60F where an enzyme concentration of 5 nM was used. For the determination of the *K*_m_ for H_2_O_2_, 20 nM enzyme concentration was employed for all protein variants. For the *K*_m_ determination of the corresponding substrate, the second substrate was utilized in its saturation concentration. The velocity quantification was achieved in the linear range of the 4-nitrocatechol using the corrected ε_425nm, corr_ = 10,870 M^–1^ cm^–1^ by applying automated path length correction in the microtiter plate. The nonlinear regression using the Michaelis–Menten model was performed with the aid of SigmaPlot (Version 14.0, Germany) yielding kinetic parameters *v*_max_, *K*_m_, and *R*^2^.

### Thermostability Measurements

Thermostability measurements were performed as described before.^32^

### Initial Oxyfunctionalization Comparison between *Mth*UPO and the Best-Performing Variants from the NBD Assay

For the identification of the best-performing variant toward the hydroxylation of naphthalene and its derivatives, an initial approach with the direct addition of H_2_O_2_ (without a syringe pump setup) was chosen. The following conditions were adjusted: 500 nM *Mth*UPO variant, 1 mM H_2_O_2_, 1 mM of the corresponding substrate (see Figures S6–S8), 100 mM KPi pH 7, 1 h at 25 °C. The reaction was quenched by the addition of 400 μL of *n*-hexane with benzyl alcohol as the internal standard. The corresponding samples were analyzed in the GC–MS scan mode, and the products were identified by library comparison. For quantification and product confirmation, the syringe pump setup was utilized.

### Bioconversion of Naphthalene and Naphthalene Derivatives Using a Syringe Pump System

With a syringe pump system, the product formation could be increased compared to the direct addition of H_2_O_2_ (initial approach). The conditions were adjusted for each reaction setup and are shown in Table S7. For the H_2_O_2_ addition, a programmable syringe pump from Chemyx Inc. (Model: Fusion 101R, Stafford, US) was utilized. The reaction and addition were performed under continuous stirring at room temperature. The extraction was accomplished by the addition of 400 μL of EtOAc (containing 1 mM 4-octanone as an internal standard) at the end of the reaction. After 30 s of vortexing, the organic layer was transferred for GC–MS analysis.

### Bioconversion of Indane and 1,2,3,4-Tetrahydronaphthalene

For the bioconversion of these substrates, no syringe pump system was needed. The reaction was started by the addition of H_2_O_2_. The hydroxylation and overoxidation to the ketone were performed under the following conditions: 100 nM L60F, 1 mM indane, 1 mM H_2_O_2_, 100 mM KPi pH 7, 5% acetone (*v*/*v*), 1 h at 25 °C in triplicates. For the hydroxylation of 1,2,3,4-tetrahydronaphthalene, the conditions were adapted from the indane bioconversion. The best-performing overoxidation of 1,2,3,4-tetrahydronaphthalene to the α-tetralone was accomplished by *Mth*UPO F59Q/L60M/S159G/F154A with a final concentration of 2 mM H_2_O_2_. The extraction was achieved by the addition of 400 μL of EtOAc (containing 1 mM 4-octanone as an internal standard) at the end of the reaction. After 30 s of vortexing, the organic layer was transferred for GC–MS analysis. Quantification of the products was carried out on an SH-Rxi-5Sil MS column under the previously mentioned GC–MS conditions. The ee determination was carried out on a Lipodex E column.

### Homology Model and MD Simulations

The homology model for the *Mth*UPO structure (245 AA, + TwinStrep-GFP11 tag) has been constructed based on the solved crystal structure of the UPO from *M. rotula* (*Mro*UPO, PDB: 5FUJ, 34% identityand 46% similarity) using the homology server SWISS-MODEL.^48^ The resulting homology model has been further refined with extensive MD simulations based on five independent replicas of 1,000 ns each, accumulating a total of 5 μs of simulation time. MD simulations in explicit water were performed using the AMBER18 package.^49^ Parameters for the different substrates (NBD, 2-methylnaphthalene, 1-methylnaphthalene, 2-methoxynaphthalene, and indane) were generated within the antechamber^50^ module in AMBER18 package using the general AMBER force field (gaff),^51^ with partial charges set to fit the electrostatic potential generated at the B3LYP/6-31G(d) level using the RESP model.^52^ The charges were calculated according to the Merz–Singh–Kollman scheme^53,54^ using the Gaussian 09 package. Parameters for the heme Cpd I and the axial Cys were taken from reference.^55^ The protein was solvated in a pre-equilibrated cubic box with a 12 Å buffer of TIP3P^56^ water molecules using the AMBER18 leap module, resulting in the addition of ∼17,500 solvent molecules. The systems were neutralized by the addition of explicit counterions (Na^+^ and Cl^–^). All subsequent calculations were carried out using the AMBER force field 14 Stony Brook (ff14SB).^57^ A two-stage geometry optimization approach was performed. The first stage minimizes the positions of solvent molecules and ions imposing positional restraints on the solute by a harmonic potential with a force constant of 500 kcal mol^–1^ Å^–2^, and the second stage is an unrestrained minimization of all the atoms in the simulation cell. The systems were gently heated using six 50 ps steps, incrementing the temperature by 50 K for each step (0–300 K) under constant-volume and periodic-boundary conditions. Water molecules were treated with the SHAKE algorithm such that the angle between the hydrogen atoms was kept fixed. Long-range electrostatic effects were modeled using the particle-mesh-Ewald method.^58^ An 8 Å cutoff was applied to Lennard-Jones and electrostatic interactions. Harmonic restraints of 10 kcal·mol^–1^ were applied to the solute, and the Langevin equilibration scheme was used to control and equalize the temperature. The time step was kept at 1 fs during the heating stages, allowing potential inhomogeneities to self-adjust. Each system was then equilibrated for 2 ns with a 2 fs time step at a constant pressure of 1 atm and a temperature of 300 K without restraints. Once the systems were equilibrated in the *NPT* ensemble, production trajectories were then run under the *NVT* ensemble and periodic-boundary conditions. In particular, a total of 5000 ns for wild-type *Mth*UPO were accumulated from five independent replicas (1000 ns each); finally, a total of 750 ns from three independent replicas were accumulated for L60F and L60F/S159G/A161F variants (250 each). Consequently, an extensive conformational sampling based on long timescale MD trajectories (1000 ns) and five different MD replicas for wild-type *Mth*UPO has been carried out to refine the initial homology model. From this accumulated simulation time, the most representative structure of *Mth*UPO was obtained by clustering analysis based on the protein backbone RMSD. This representative structure was used for further MD simulations, analyses, and preparation of the other studied variants. Trajectories were processed and analyzed using the cpptraj^59^ module from Ambertools utilities.

### Docking and Protocol Used for Substrate-Bound MD Simulations

Docking calculations were performed using AutoDock Vina.^60^ Representative structures for the most populated clusters (based on backbone RMSD clustering analysis) obtained from MD simulations carried out in the apo state were used, and docking predictions were then utilized as starting points for substrate-bound MD simulations. To avoid substrate diffusion outside the enzyme active site and to sample catalytically competent binding poses, a 100 kcal·mol^–1^·Å^–2^ restraint is applied when the distances between the center of mass of the substrate and the O atom of the Fe=O in Cpd I are greater than 6 Å (for indane) or 6.7 Å (for naphthalene derivatives). No restraints were used in NBD substrate-bound simulations. The same protocol for MD simulations described above has been employed, accumulating a total of 300 ns of production trajectories from three independent replicas for all substrate-bound studies.

### Quantum Mechanics Calculations

Density functional theory calculations were carried out using Gaussian 09.^61^ Geometry optimizations and frequency calculations were performed using the (U)B3LYP^62−64^ functional with the 6-31G(d) basis set on all atoms. The stationary points were verified as minima by a vibrational frequency analysis. Enthalpies and entropies were calculated for 1 atm and 298.15 K. Single-point energy calculations were performed using the functional (U)B3LYP with the Def2TZVP basis set on all atoms and within the CPCM polarisable conductor model (dichloromethane, ε = 8.9)^65,66^ to have an estimation of the dielectric permittivity in the enzyme active site.^67^ BDEs were calculated as the standard enthalpic change in the following process at 298 K (indane → indane^•^ + H^•^), which provides an estimation of the strength of the C–H bond under study. Different electronic states (singlet close-shell for indane and doublet for both radical species) have been considered.

## Supporting Information Available

Strategies for primer design, sequences of the utilized UPOs, kinetic plots, GC parameters and original GC chromatograms, activities and selectivities of all tested variants, reaction conditions, calibration curves, and figures of docking studies and MD simulations (PDF)
