# Supplementary material for: Accessing Chemo- and Regioselective Benzylic and Aromatic Oxidations by Protein Engineering of an Unspecific Peroxygenase
Source: ACS Catal. 2021 Jun 7;11(12):7327–38. doi: 10.1021/acscatal.1c00847 (PMC8496131; doi:10.1021/acscatal.1c00847)
Supplement: Supplementary file 1 — cs1c00847_si_001.pdf [file cs1c00847_si_001.pdf]

Supporting Information

Accessing Chemo- and Regioselective Benzylic and Aromatic Oxidations by Protein Engineering of an Unspecific Peroxygenase

Anja Knorrscheidt,[a] Jordi Soler,[c] Nicole Hünecke, [a] Pascal Püllmann, [a] Marc Garcia-Borràs*[c] and Martin J. Weissenborn*[a, b]

***Corresponding author. Email: marc.garcia@udg.edu**

***Corresponding author. Email: martin.weissenborn@ipb-halle.de**

a. Bioorganic Chemistry, Leibniz Institute of Plant Biochemistry, Weinberg 3, 06120 Halle (Saale), Germany.
b. Institute of Chemistry, Martin Luther University Halle-Wittenberg, Kurt-Mothes-Str. 2, 06120 Halle (Saale), Germany.
c. Institut de Química Computacional i Catàlisi and Departament de Química, Universitat de Girona, Carrer Maria Aurèlia Capmany 69, Girona 17003, Catalonia, Spain.

Gene and amino acid sequence of *Mth*UPO wildtype with signal peptide from *α* Galactosidase (*S.cerevisiae*) (underline) and TwinStrep-GFP11 tag (*italic*) in pAGT572_Nemo 2.0:

ATGTTTGCTTTTTATTTCTTGACTGCTTGTATTTCTTTGAAAGGTGTTTTTGGAGCAGGTTTTGACACTTGGTCAC
CACCTGGACCCTATGATGTTAGGGCTCCTTGTCCGATGTTGAATACATTGGCTAATCATGGTTTCTTACCACATG
ATGGCAAAGATATTACTCGTGAACAGACAGAAAACGCCTTGTTCGAAGCATTGCACATCAACAAAACCTTAGC
CAGCTTTCTGTTTGACTTTGCATTAACAACGAATCCGAAGAATACCTCGACGTTTTCACTGAACGACTTAGGCA
ATCACAACATTTTGGAACATGATGCATCACTAAGTAGGGCTGATGCGTACTTTGGGAATGTTCTACAGTTCAAT
CAAACTGTCTTTGATGAGACTAAAACCTACTGGGAAGGAGATACTATTGATTTGAGAATGGCAGCCAAAGCTA
GACTAGGTAGAATCAAGACATCTCAAGCTACTAATCCAACGTATTCCATGTCGGAATTAGGAGATGCTTTCACA
TATGGGGAATCTGCTGCGTATGTGGTAGTGTTAGGTGACAAAGAGTCTCGTACTGTCAAAAGATCCTGGGTTG
AATGGTTCTTCGAACATGAGCAACTTCCTCAACATCTTGGTTGGAAAAGACCAGCAGCATCCTTCGAAGAAGA
AGATCTGAACTCATCAATGGAGGAGATTGAGAAGTACACCAAGGAATTGGAAGGTAGCAACTCTACAAGTGG
TAGTCAAAAGCATAGAAGGAGACTTCCAAGAAGAAGAGCTCACTTTGGCTTTTCGGGTGGTTCTGCTTGGTCA
CATCCACAATTTGAAAAAGGTGGAGGTTCAGGTGGAGGT*TCGGGTGGTTCTGCTTGGTCACATCCACAATTTG*
AAAAAGATGGTGGTTCTGGTGGTGGTTCTACTAGTCGTGATCATATGGTTCTTCATGAATATGTTAATGCTGCT
GGTATTACTTGA

MFAFYFLTACISLKGVFGAGFDTWSPPGPYDVRAPCPMLNTLANHGFLPHDGKDITREQTENALFEALHINKTLASF
LFDFALTTNPKNTSTFSLNDLGNHNILEHDASLSRADAYFGNVLQFNQTVFDETKTYWEGDTIDLRMAAKARLGRIK
TSQATNPTYSMSELGDAFTYGESAAYVVVLGDKESRTVKRSWVEWFFEHEQLPQHLGWKRPAASFEEEDLNSSME
EIEKYTKELEGSNSTSGSQKHRRRLPRRRAHFGF*SGGSAWSHPQFEKGGGSGGGSGGSAWSHPQFEKDGGSGGG*
STSRDHMVLHEYVNAAGIT

Primer design. All oligonucleotides were purchased in the purification grad “desalted” at Eurofins (Ebersberg, DE). The exemplary primer design of the single saturation mutagenesis library is illustrated in Table S1.

Table S1 Exemplary primer list for the Golden Mutagenesis approach of the single saturation library at position L60 (computer designed primers by the Golden Mutagenesis tool: https://msbi.ipb-halle.de/GoldenMutagenesisWeb/)

Name	Sequence (5' → 3')
Fragment I_for	TTGGTCTCAAATGTTTGCTTTTTATTTCTTGACTGCTTGT
Fragment I_NDT_rev	TTGGTCTCTGTCAAAAHNAAAGCTGGCTAAGGTTTTGTTGATG
Fragment I_VHG_rev	TTGGTCTCTGTCAAACDBAAAGCTGGCTAAGGTTTTGTTGATG
Fragment I_TGG_rev	TTGGTCTCTGTCAAACCAAAAGCTGGCTAAGGTTTTGTTGATG
Fragment II_for	TTGGTCTCATGACTTTGCATTAACAACGAATCCGAAGAAT
Fragment II_rev	TTGGTCTCAGGCAACTTTTGCGGCGGCCTTCCCGGACGCGCATCTTGGTG

Table S2 Instrumental parameters for GC-MS measurements

Products	GC-MS	Column	Temperature program
1,4-Naphthoquinone (*m/z* 158)	Achiral	SH-Rxi-5Sil MS	40 °C<br>7 °C/min to 190 °C<br>100 °C/min to 300 °C hold 10 min
Silylated 2-Hydroxy-1,4-naphthoquinone (Lawsone, *m/z* 231)	Achiral	SH-Rxi-5Sil MS	70 °C<br>20 °C/min to 270 °C<br>100 °C/min to 300 °C hold 4 min
2-Methyl-1,4-naphthoquinone (Menadione, *m/z* 172)	Achiral	SH-Rxi-5Sil MS	40 °C<br>7 °C/min to 190 °C<br>100 °C/min to 300 °C hold 10 min
2-Naphthalenemethanol (*m/z* 158)<br>2-Naphthaldehyde (*m/z* 156)	Achiral	SH-Rxi-5Sil MS	40 °C<br>7 °C/min to 190 °C<br>100 °C/min to 300 °C hold 10 min
5-Methyl-1,4-naphthoquinone (*m/z* 172)	Achiral	SH-Rxi-5Sil MS	40 °C<br>7 °C/min to 190 °C<br>100 °C/min to 300 °C hold 2 min
6-Methoxy-1,4-naphthoquinone (*m/z* 188)	Achiral	SH-Rxi-5Sil MS	40 °C<br>7 °C/min to 190 °C<br>100 °C/min to 300 °C hold 10 min
Silylated 6-Bromo-1,4-naphthoquinone (*m/z* 384)	Achiral	SH-Rxi-5Sil MS	70 °C<br>20 °C/min to 270 °C<br>100 °C/min to 300 °C hold 4 min
1-Hydroxyindan (*m/z* 133)<br>1-Indanol (*m/z* 132)	Achiral (quantification)	SH-Rxi-5Sil MS	40 °C<br>7 °C/min to 190 °C<br>100 °C/min to 300 °C hold 2 min
	Chiral (*ee* determination)	Lipodex E	95 °C<br>0.5 °C/min to 110 °C<br>100 °C/min to 200 hold 2 min
1,2,3,4-Tetrahydro-1-naphthol (*m/z* 148)<br>α-Tetralone (*m/z* 146)	Achiral (quantification)	SH-Rxi-5Sil MS	40 °C<br>7 °C/min to 190 °C<br>100 °C/min to 300 °C hold 2 min
	Chiral (*ee* determination)	Lipodex E	75 °C<br>1 °C/min to 120 °C<br>100 °C/min to 200 hold 2 min

Table S3 Overview of generated libraries.

Library #	Mutagenesis approach	Degeneracy	Number of screened variants	Statistical library coverage [%][a]
1	Single saturation (9 positions)	22c-trick	For each position: 88<br>In total: 792	98
2	Double saturation (5 grouped positions)	NDT	For each position: 440<br>In total: 2200	95
3	Recombination (864 possible combinations)	Best performing variants from library 1/2	In total: 2376	94

[a] The statistical library coverage was calculated using the equation for fractional library completeness[1]: $L = - V \ln(1-F)$; L – Number of screened variants, V – number of possible variants, F – library coverage

Table S4 Overview of secreted variants within the single saturated mutagenesis libraries.

Saturated position	Secreted variants [%]
L56	52
F59	93
L60	75
L86	76
F154	81
T155	83
S159	83
A161	72
L206	69

Table S5 Catalytic activity of purified *Mth*UPO variants for the hydroxylation of NBD.[a]

Catalyst	T_m [°C]	TOF [min^{-1}]	TON	Conversion [%]
*Mth*UPO WT	63.5 ± 0.09	72	4340	29
*Mth*UPO L60M		83	5000	33
*Mth*UPO L60Q		94	5680	38
*Mth*UPO L60F	55.7 ± 0.44	194	11610	77
*Mth*UPO F59Q		72	4360	29
*Mth*UPO L60F/F154I		90	5380	36
*Mth*UPO L60F/F154V		87	5200	35
*Mth*UPO I52/A57I		71	4290	29
*Mth*UPO S159N/A161F		75	4510	30
*Mth*UPO S159G/A161I		75	4520	30
*Mth*UPO L60F/S159G/A161I	56.9 ± 0.18	198	11850	79
*Mth*UPO L60F/S159G/A161F		210	12590	84
*Mth*UPO F59Q/L60M/S159G/F154A	54.7 ± 0.41	192	11540	77
*Mth*UPO F59Q/L60F/A161I		199	11950	80
*Mth*UPO F59Q/L60F/S159G	55.8 ± 1.26	379	22760	76

TOF = turnover frequency, TON = turnover number, standard deviation < 3.2 %, [a] Reaction conditions: 20 nM *Mth*UPO variant, 300 µM NBD, 1 mM H_2O_2, 100 mM KPi buffer (pH 7), 5 % acetone (v/v), measurement conditions: absorbance was measured at 425 nm for one hour in triplicates, values were calculated with the corrected extinction coefficient of 10870 $M^{-1}*cm^{-1}$, [b] reaction time over night, [c] 10 nM *Mth*UPO variant.

Table S6 Kinetic parameters of the *Mth*UPO variants for H_2O_2 as a substrate.[a]

Catalyst	K_m [mM]	k_{cat} [s^{-1}]	k_{cat}/K_m [$M^{-1}s^{-1}$] x 10^4
*Mth*UPO WT	0.45 ± 0.10	5.2 ± 0.10	1.3
*Mth*UPO L60F	0.93 ± 0.150	1.1	0.1
*Mth*UPO L60F/S159G/A161F	1.04 ± 0.10	93.4	9.0
*Mth*UPO F59Q/L60M/S159G/F154A	2.63 ± 0.55	70.6	2.7
*Mth*UPO F59Q/L60F/S159G	3.13 ± 0.43	24.7	0.8

Standard deviation < 20 %, TOF = turnover frequency, TON = turnover number, [a] Reaction conditions: 20 nM *Mth*UPO variant, NBD saturation concentration was adjusted for each variant depending on the K_m value of NBD (see Table 2), H_2O_2 concentration was varied as depicted in Figure S3. Measurement conditions: absorbance was measured at 425 nm for one hour in triplicates, values were calculated with the corrected extinction coefficient of 10870 $M^{-1}*cm^{-1}$.

Table S7 Overview of reaction conditions for the hydroxylation of naphthalene and naphthalene derivatives.

Product	Catalyst	Reaction conditions
1a 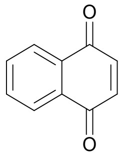	MthUPO F59Q/L60F/S159G	1 mM naphthalene, 4 mM H_2O_2, 50 nM catalyst, 100 mM KPi pH 7, 5 % (v/v) acetone, V_{total} = 400 µl, syringe pumpe addition of a 16 mM H_2O_2 stock solution (100 µl stock solution, 100 µl/h), 30 min additional stirring
1b 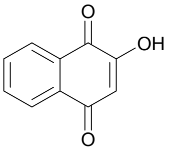	*Mth*UPO F59Q/L60F/S159G	1 mM naphthalene, 5 mM H_2O_2, 500 nM catalyst, 100 mM KPi pH 7, 5 % (v/v) acetone, V_{total} = 400 µl, syringe pumpe addition of a 10 mM H_2O_2 stock solution (200 µl stock solution, 200 µl/h), additional stirring overnight
2a 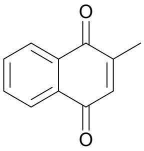	*Mth*UPO L60F/S159G/A161F	1 mM 2-methylnaphthalene, 4 mM H_2O_2, 100 nM catalyst, 100 mM KPi pH 7, 5 % (v/v) acetone, V_{total} = 400 µl, syringe pumpe addition of a 8 mM H_2O_2 stock solution (200 µl stock solution, 100 µl/h), 1 additional stirring
2b 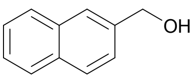 2c 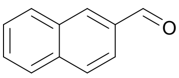	*Mth*UPO L60F	1 mM 2-methylnaphthalene, 4 mM H_2O_2, 500 nM catalyst, 100 mM KPi pH 7, 5 % (v/v) acetone, V_{total} = 400 µl, syringe pumpe addition of a 8 mM H_2O_2 stock solution (200 µl stock solution, 100 µl/h), 30 min additional stirring
3a 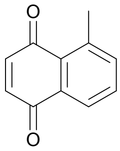	*Mth*UPO L60F/S159G/A161F	1 mM 1-methylnaphthalene, 4 mM H_2O_2, 500 nM catalyst, 100 mM KPi pH 7, 5 % (v/v) acetone, V_{total} = 400 µl, syringe pumpe addition of a 8 mM H_2O_2 stock solution (200 µl stock solution, 200 µl/h)
4a 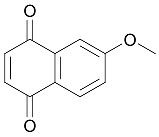	*Mth*UPO L60F/S159G/A161F	1 mM 2-methoxynaphthalene, 4 mM H_2O_2, 500 nM catalyst, 100 mM KPi pH 7, 5 % (v/v) acetone, V_{total} = 400 µl, syringe pumpe addition of a 8 mM H_2O_2 stock solution (200 µl stock solution, 200 µl/h), additional stirring overnight
5a 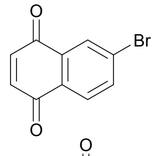 5b 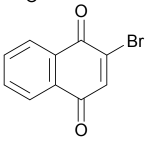	*Mth*UPO L60F/S159G/A161F	1 mM 2-bromonaphthalene, 4 mM H_2O_2, 500 nM catalyst, 100 mM KPi pH 7, 5 % (v/v) acetone, V_{total} = 400 µl, syringe pumpe addition of a 8 mM H_2O_2 stock solution (200 µl stock solution, 200 µl/h), 1 h additional stirring

Standard deviation of triplicates <5.2 %.

Table S8 Enantioselectivity of the evolved variants towards the hydroxylation of indane and 1,2,3,4-tetrahydronaphthalene.

Product	Catalyst	Configuration	*ee* [%]
5a 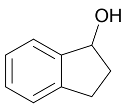	*Mth*UPO WT	*R*	84
	*Mth*UPO L60F	*R*	95
	*Mth*UPO L60F/S159G/A161F	*S*	14
	*Mth*UPO F59Q/L60M/S159G/F154A	*S*	14
	*Mth*UPO F59Q/L60F/S159G	*R*	91
6a 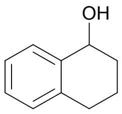	*Mth*UPO WT	*R*	45
	*Mth*UPO L60F	*R*	74
	*Mth*UPO L60F/S159G/A161F	*R*	6
	*Mth*UPO F59Q/L60M/S159G/F154A	*S*	19
	*Mth*UPO F59Q/L60F/S159G	*R*	50

Table S9 Activities of the evolved variants towards the hydroxylation of indane and 1,2,3,4-tetrahydronaphthalene.

Product	Catalyst	Turnover number
5a 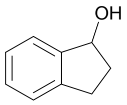	*Mth*UPO WT	7140
	*Mth*UPO L60F	8160
5b 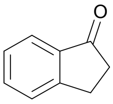	*Mth*UPO WT	260
	*Mth*UPO L60F	450
6a 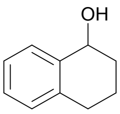	*Mth*UPO WT	980
	*Mth*UPO L60F	860
6b 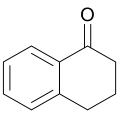	*Mth*UPO WT	350
	*Mth*UPO F59Q/L60M/S159G/F154A	440

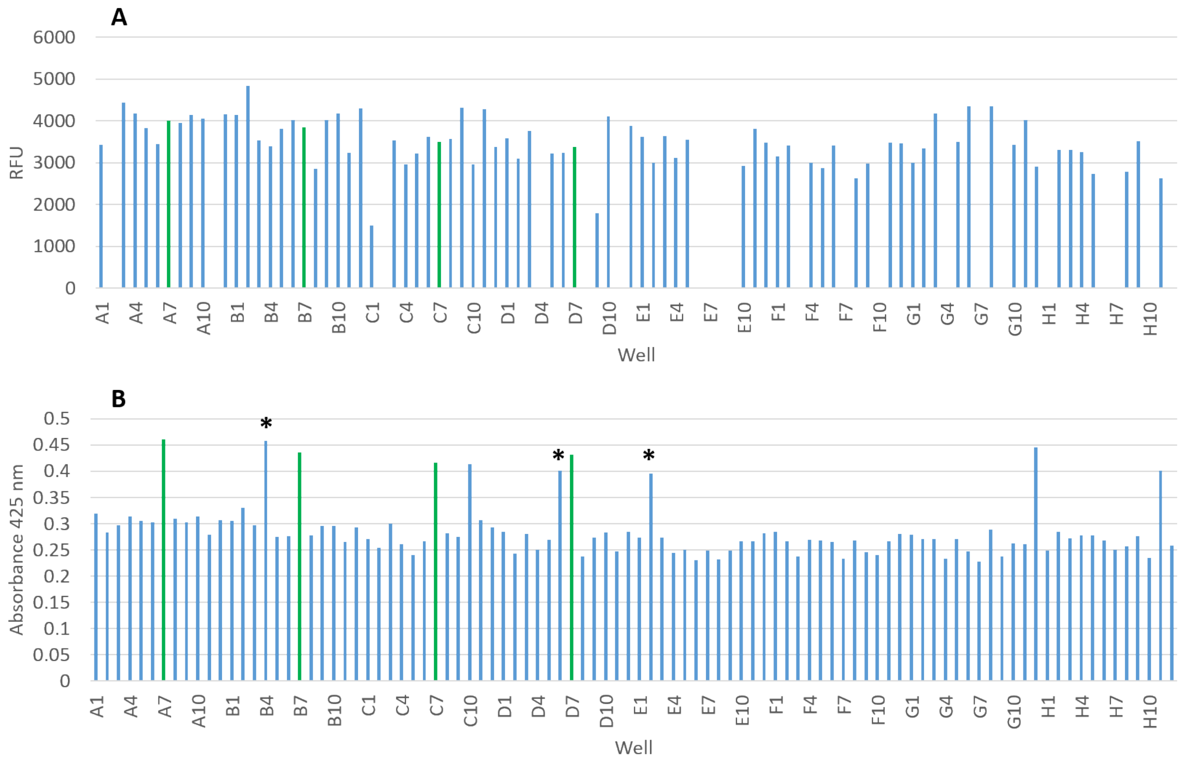

Figure S1 Screening results of saturated position F154, A) split-GFP results depicts secreted variants whereas B) illustrates the active variants within the NBD assay, green = WT as control, * = sequenced variants which confirmed the WT.

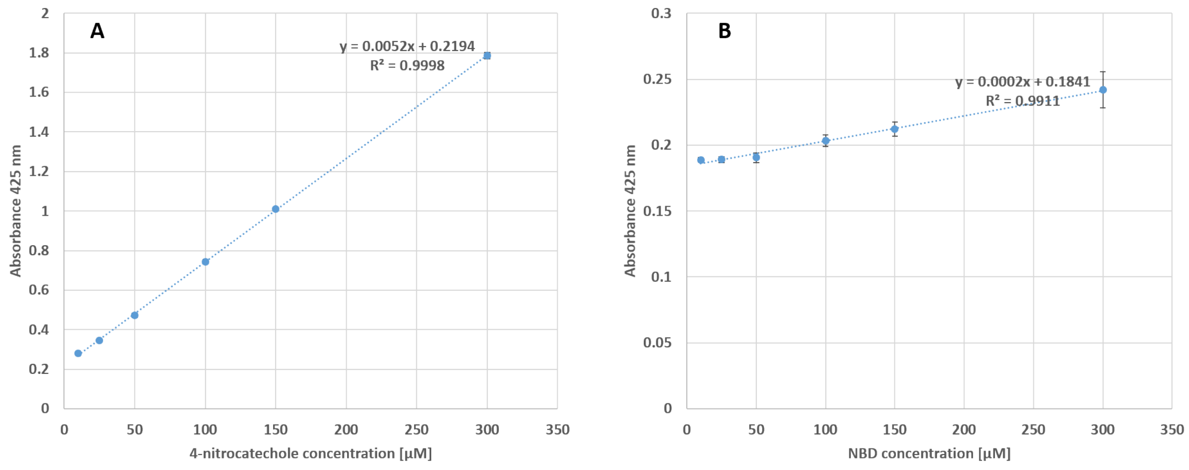

Figure S2. Calibration curves of A) 4-nitrocatechol and B) NBD under NBD-assay reaction conditions.

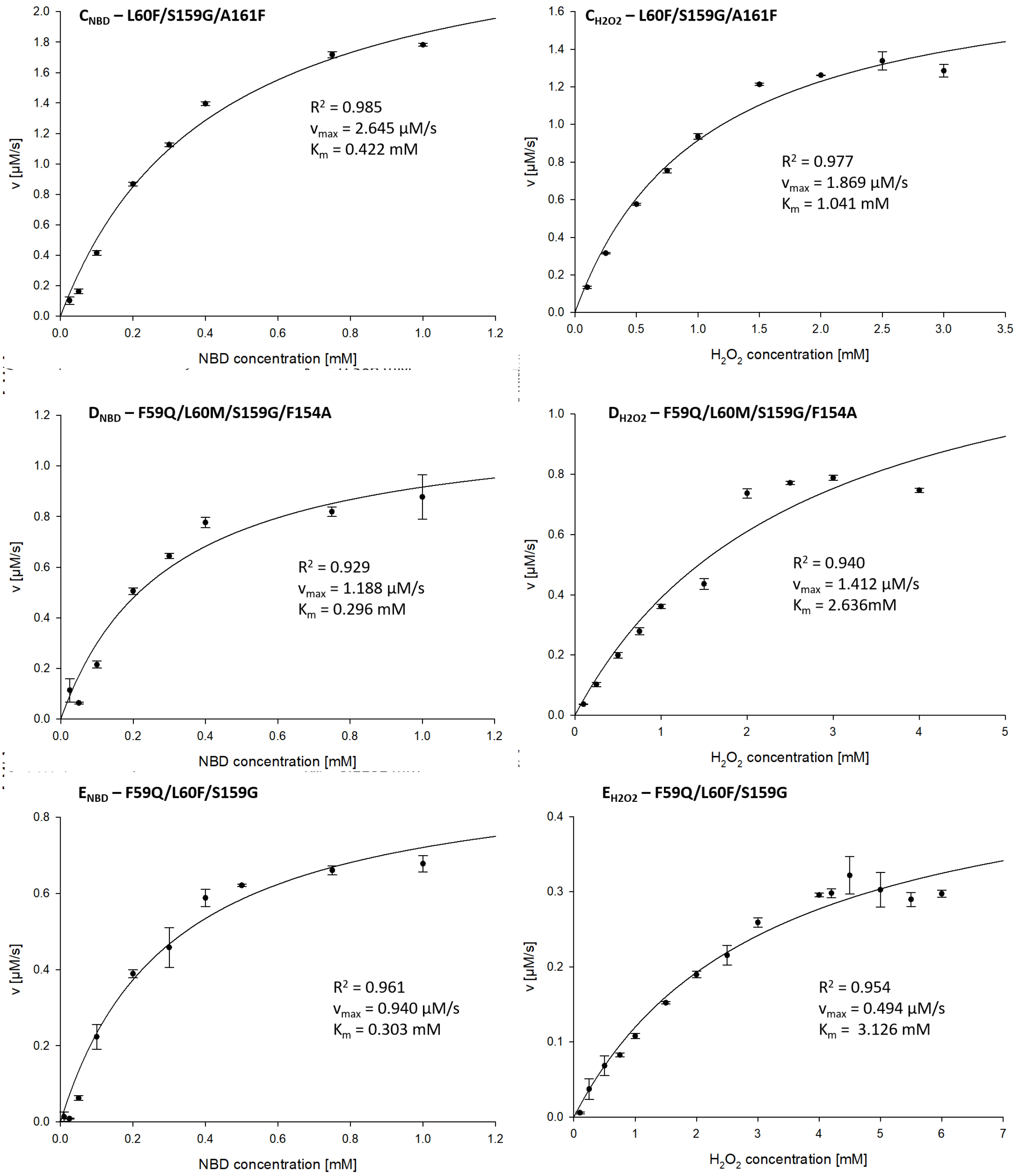

Figure S3. Michaelis-Menten plots of the best performing variants and their calculated kinetic parameters.

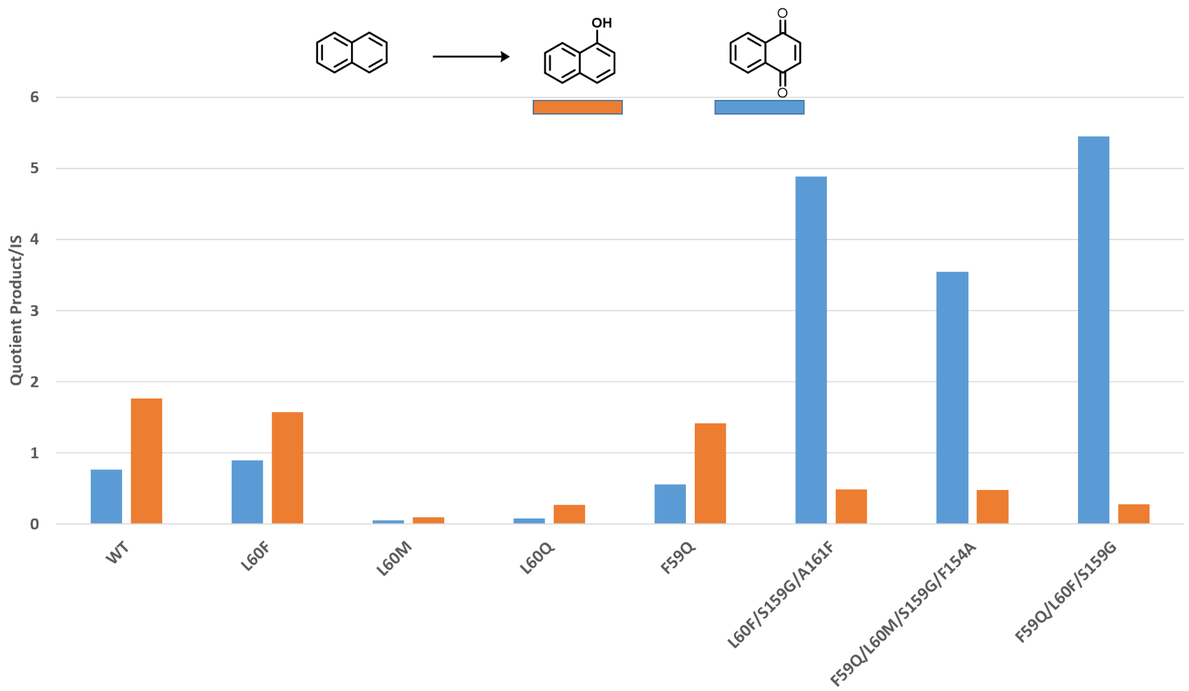

Figure S4 Product distribution of *Mth*UPO variants towards the bioconversion of naphthalene.

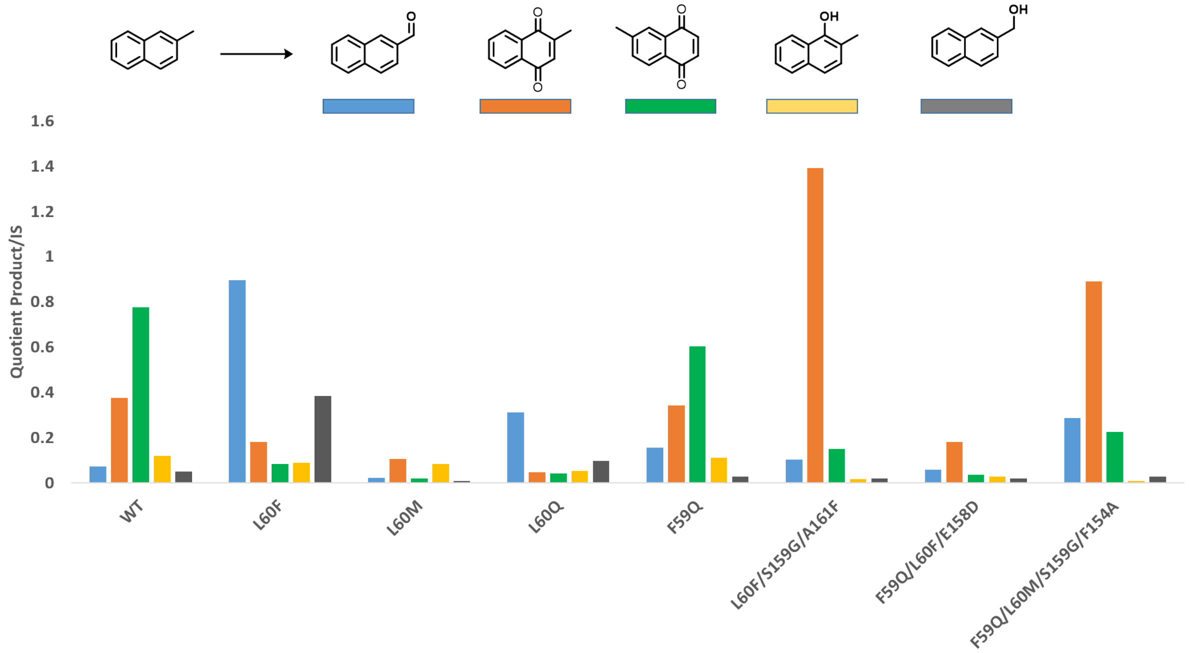

Figure S5 Product distribution of *Mth*UPO variants towards the bioconversion of 2-methylnaphthalene.

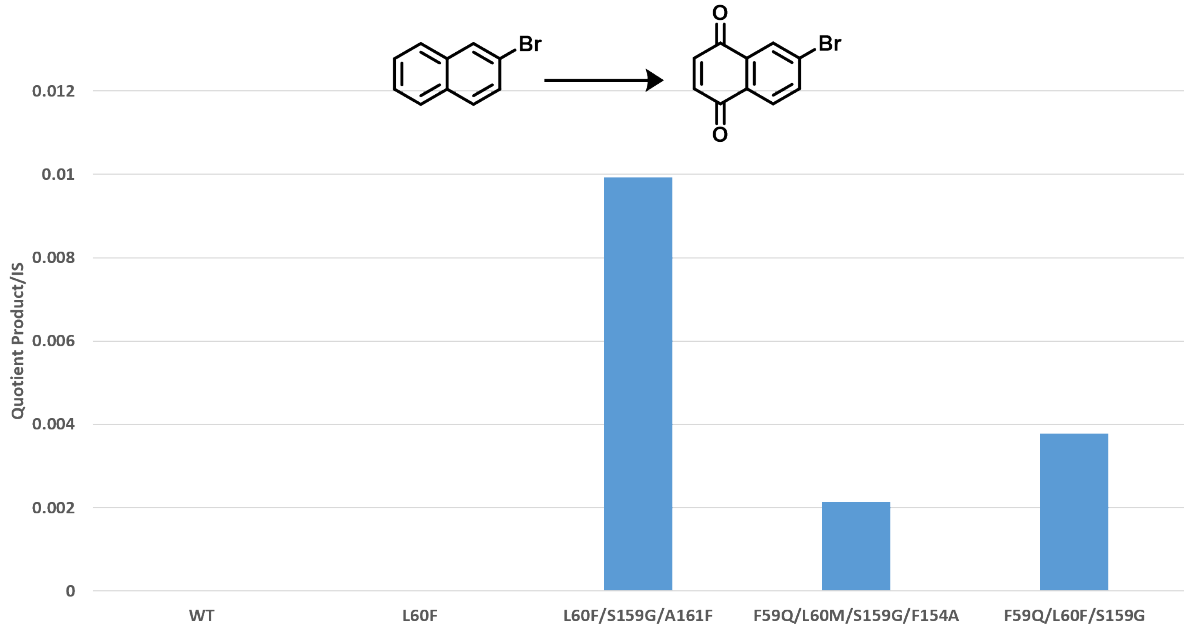

Figure S6 Product distribution of *Mth*UPO variants towards the bioconversion of 2-bromonaphthalene.

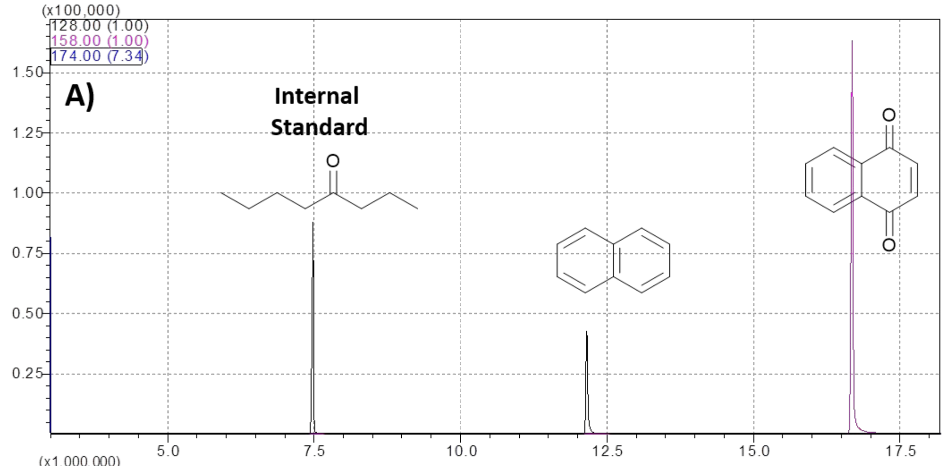
(x100,000)
128.00 (1.00)
158.00 (1.00)
174.00 (7.34)
A)
Internal
Standard
O
O
O

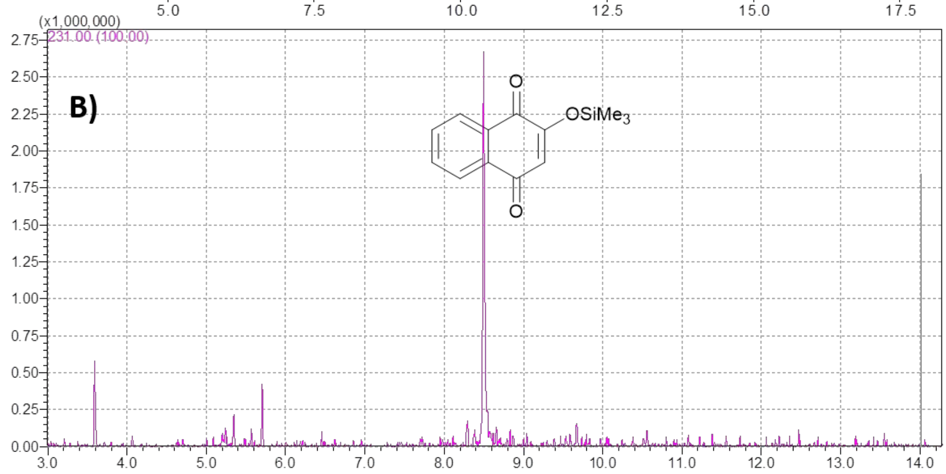
(x1,000,000)
231.00 (100.00)
B)
O
OSiMe3
O

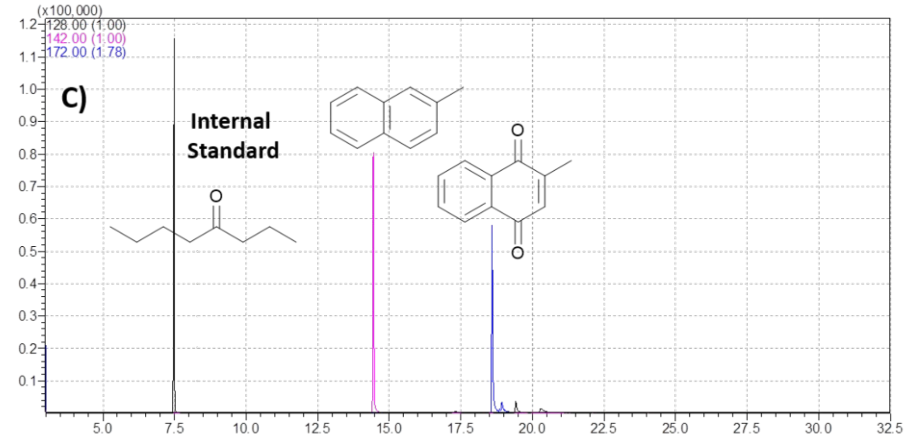
(x100,000)
128.00 (1.00)
142.00 (1.00)
172.00 (1.78)
C)
Internal
Standard
O
O
O

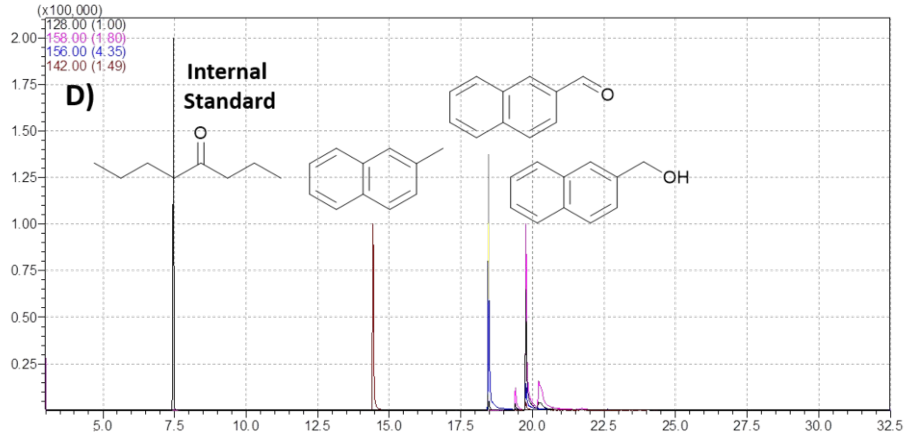
(x100,000)
128.00 (1.00)
158.00 (1.80)
156.00 (4.35)
142.00 (1.49)
D)
Internal
Standard
O
O
OH

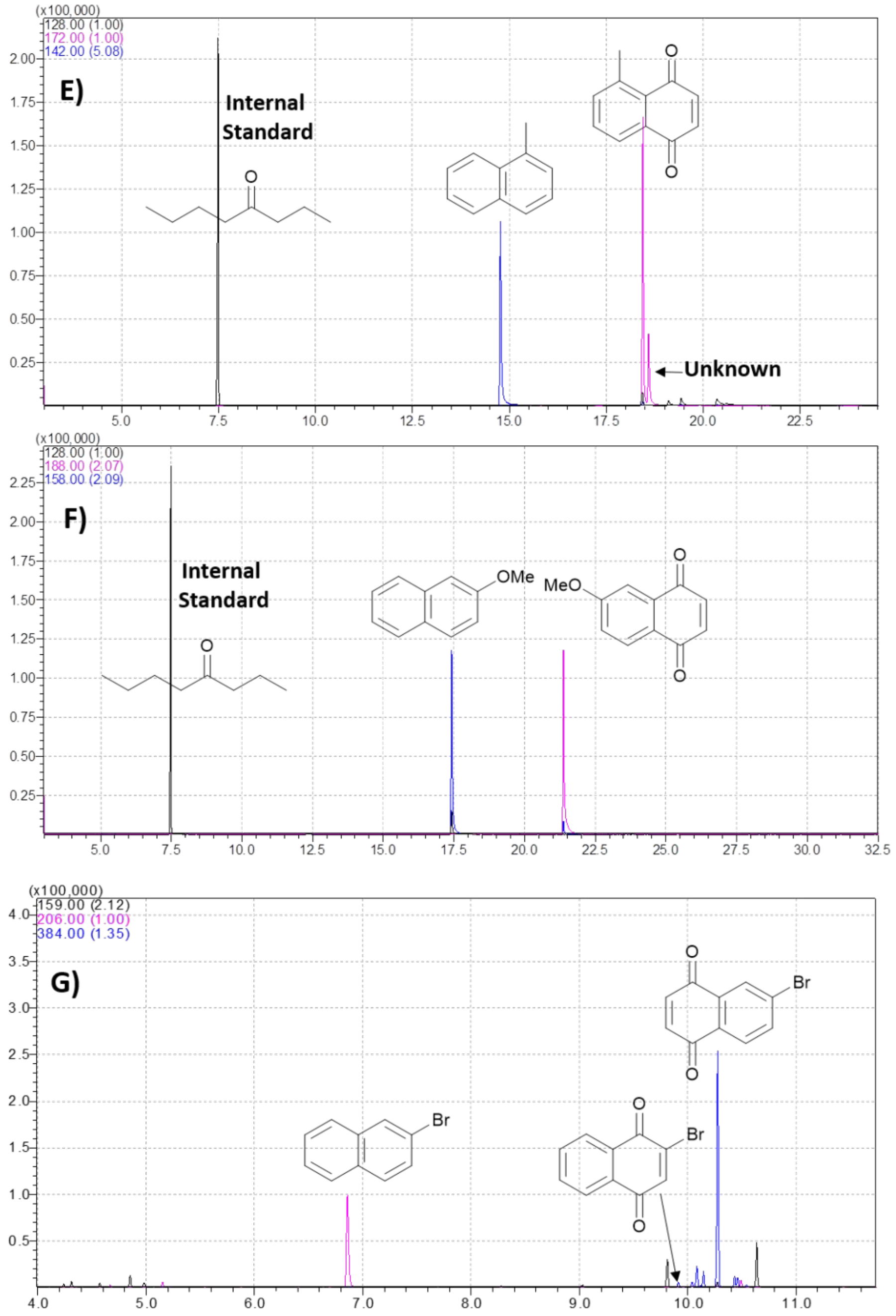

Figure S7 GC-MS chromatogram (in selected ion monitoring mode with the depicted *m/z* traces) of the later described *Mth*UPO variant of naphthalene and naphthalene derivatives, A) F59Q/L60F/S159G with naphthalene to 1,4-naphthoquinone, B) F59Q/L60F/S159G with naphthalene to Lawsone product, C) L60F/S159G/A161F with 2-methylnaphthalene to vitamin K_3, D) L60F with 2-methylnaphthalene to 2-naphthalenemethanol and *β*-naphthaldehyde, E) L60F/S159G/A161F with 1-methylnaphthalene to 5-methyl-1,4-naphthoquinone, F) L60F/S159G/A161F with 2-methoxynaphthalene to 6-methoxy-1,4-naphthoquinone and G) L60F/S159G/A161F with 2-bromonaphthalene to 6-bromo-1,4-naphthoquinone and 2-bromo-1,4-naphthoquinone.

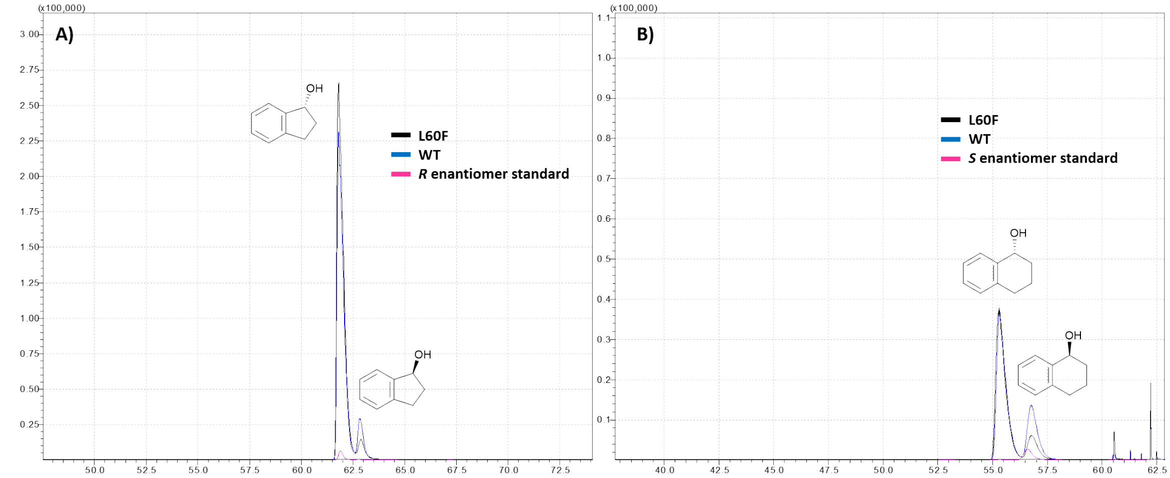

Figure S8 Chiral GC-MS chromatogram of the bioconversion of a) indane and b) 1,2,3,4-tetrahydronaphthalene to the corresponding hydroxylated products.

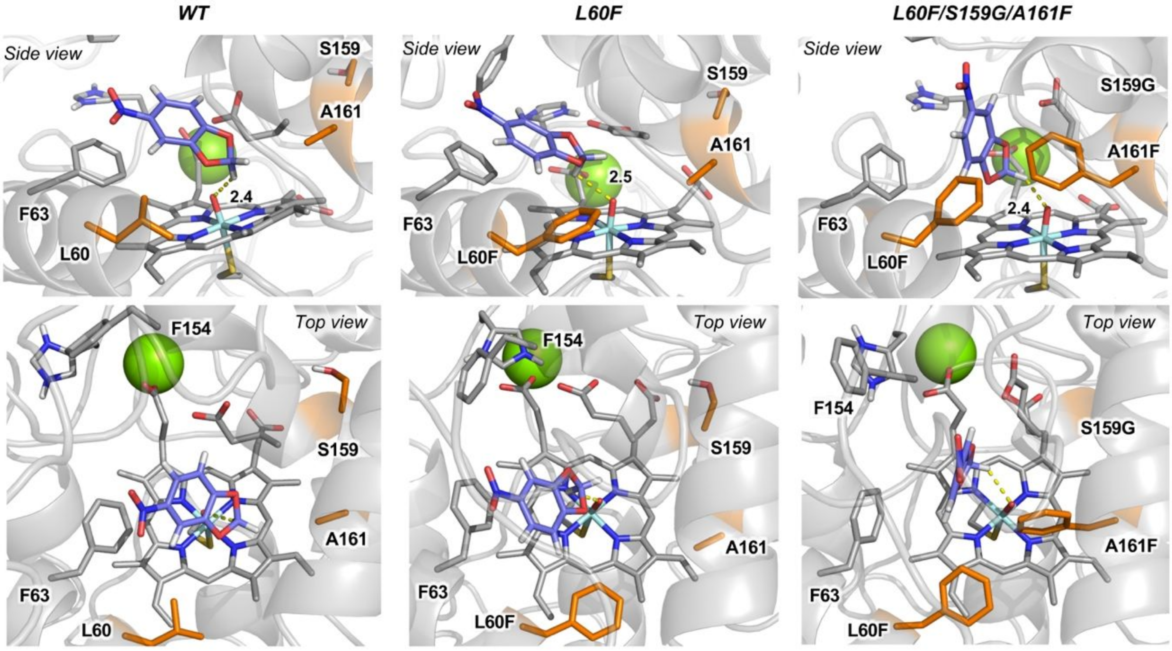

Figure S9. Evolution of NBD catalytically relevant binding modes in wildtype, L60F and L60F/S159G/A161F variants as observed from MD simulations (see Figure S10). Mutated positions are highlighted in orange. NBD explores substantially different near attack conformations (NACs) in each variant due to the new introduced mutations. L60F displaces NBD from a more buried binding pose in wildtype, to a new binding mode that increases the aromatic interactions with residues F63 and newly introduced L60F. Finally, A161F and S159G mutations led to a significantly reduced active site that forces NBD to explore a new binding mode, perpendicular to the haem, that facilitates its interaction with the catalytic Fe=O species (see also Figure S10).

A)

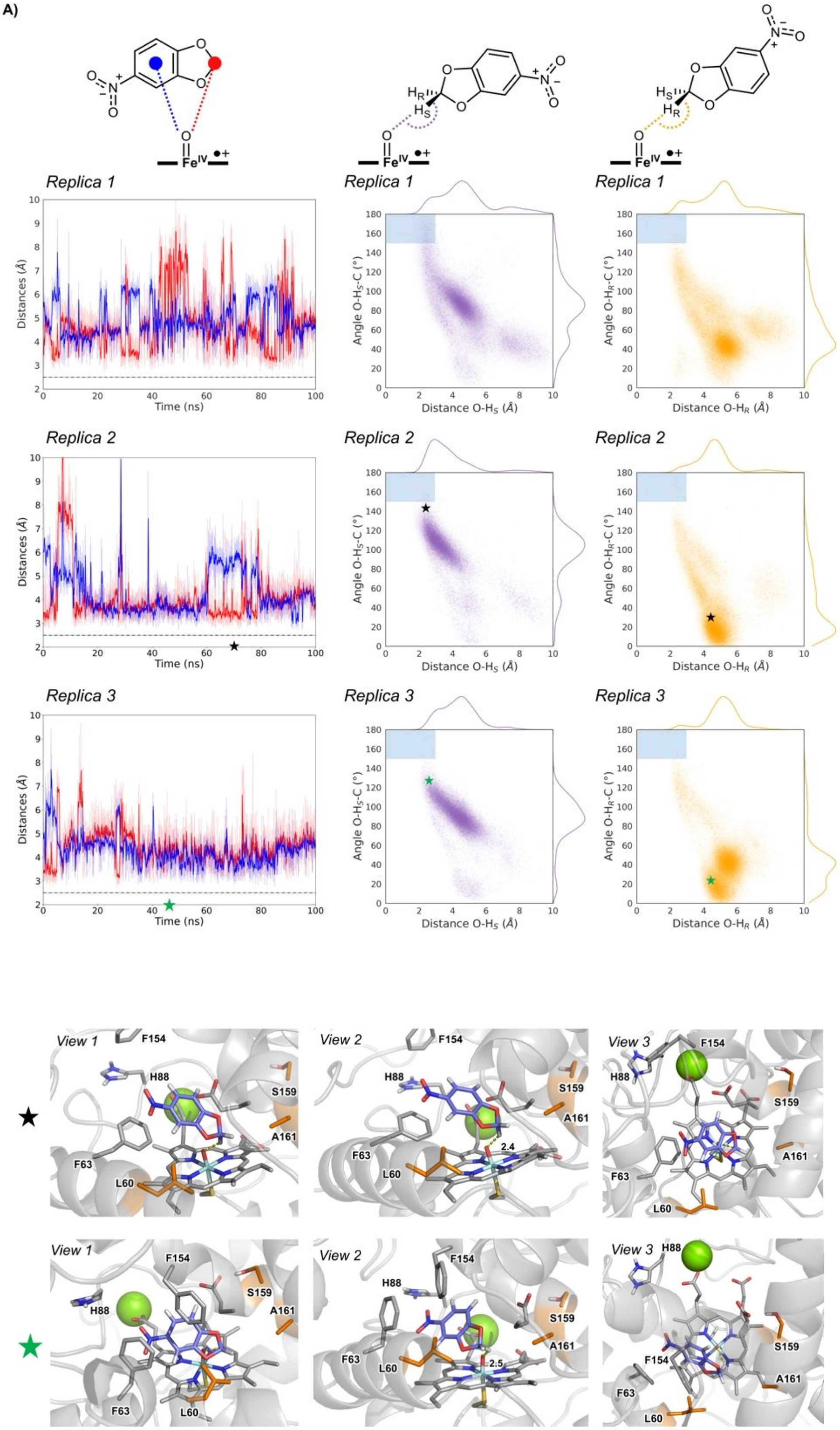

Replica 1
Replica 2
Replica 3
Distances (Å)
Time (ns)
Angle O-H$_S$-C (°)
Distance O-H$_S$ (Å)
Angle O-H$_R$-C (°)
Distance O-H$_R$ (Å)
View 1
View 2
View 3
F154
H88
S159
A161
F63
L60
2.4
2.5

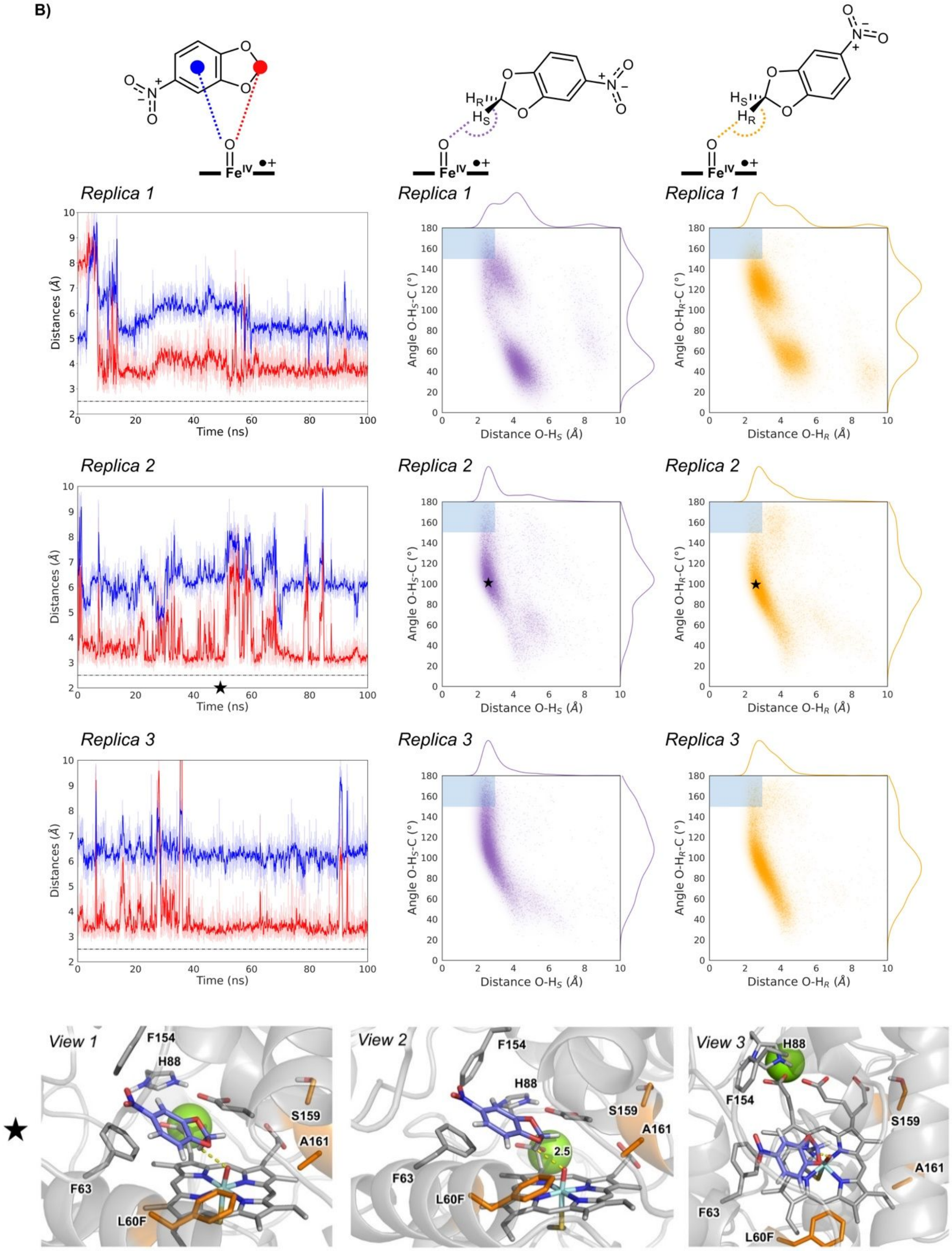

B)
Fe^IV •+
Replica 1
Replica 2
Replica 3
Distances (Å)
Time (ns)
Angle O-H_S-C (°)
Distance O-H_S (Å)
Angle O-H_R-C (°)
Distance O-H_R (Å)
View 1
View 2
View 3
F154
H88
S159
A161
F63
L60F
2.5

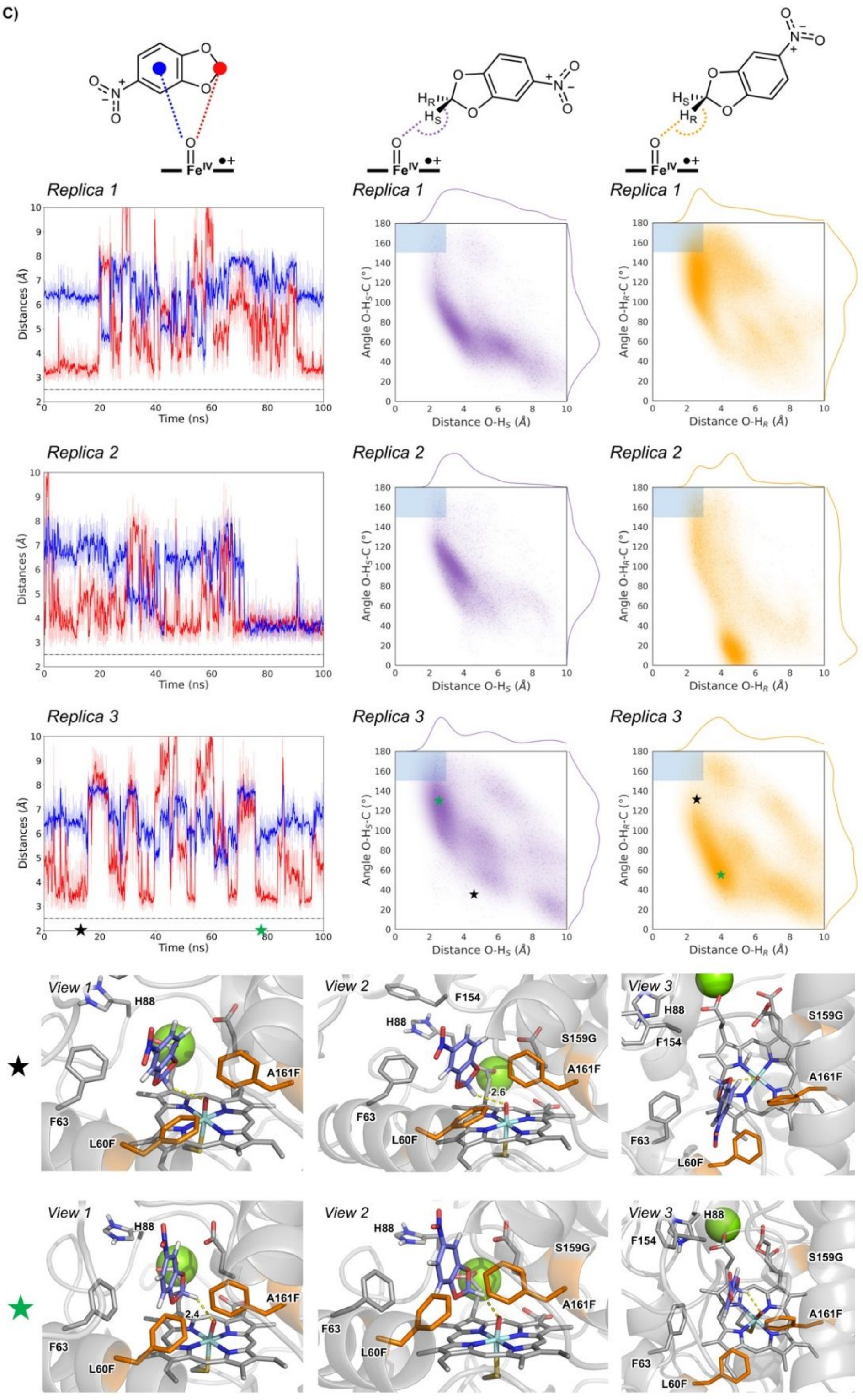

Figure S10. Analysis of NBD binding modes through three independent MD replicas in: **A**) wildytpe; **B**) L60F variant; and **C**) L60F/S159G/A161F variant. Key distances relevant for hydroxylation and aromatic oxidation are monitored along MD simulations, as described in the schemes. Fe=O – H(CH) distance and angle of attack (O-H-C) are used as geometric parameters to characterise near attack conformations for effective C-H hydroxylation in heat maps. Representative snapshots from MD trajectories (highlighted with a "star" symbol) that describe reactive near attack conformations explored during MDs are shown. Distances and angles are given in angstroms (Å) and degrees (º), respectively. Angle vs. distance heat maps show that NBD explores more catalytically competent poses in L60F/S159G/A161F and L60F variants than in the WT, in line with the higher activity experimentally observed. The differences in the NBD binding poses in the three variants are discussed in Figure S9.

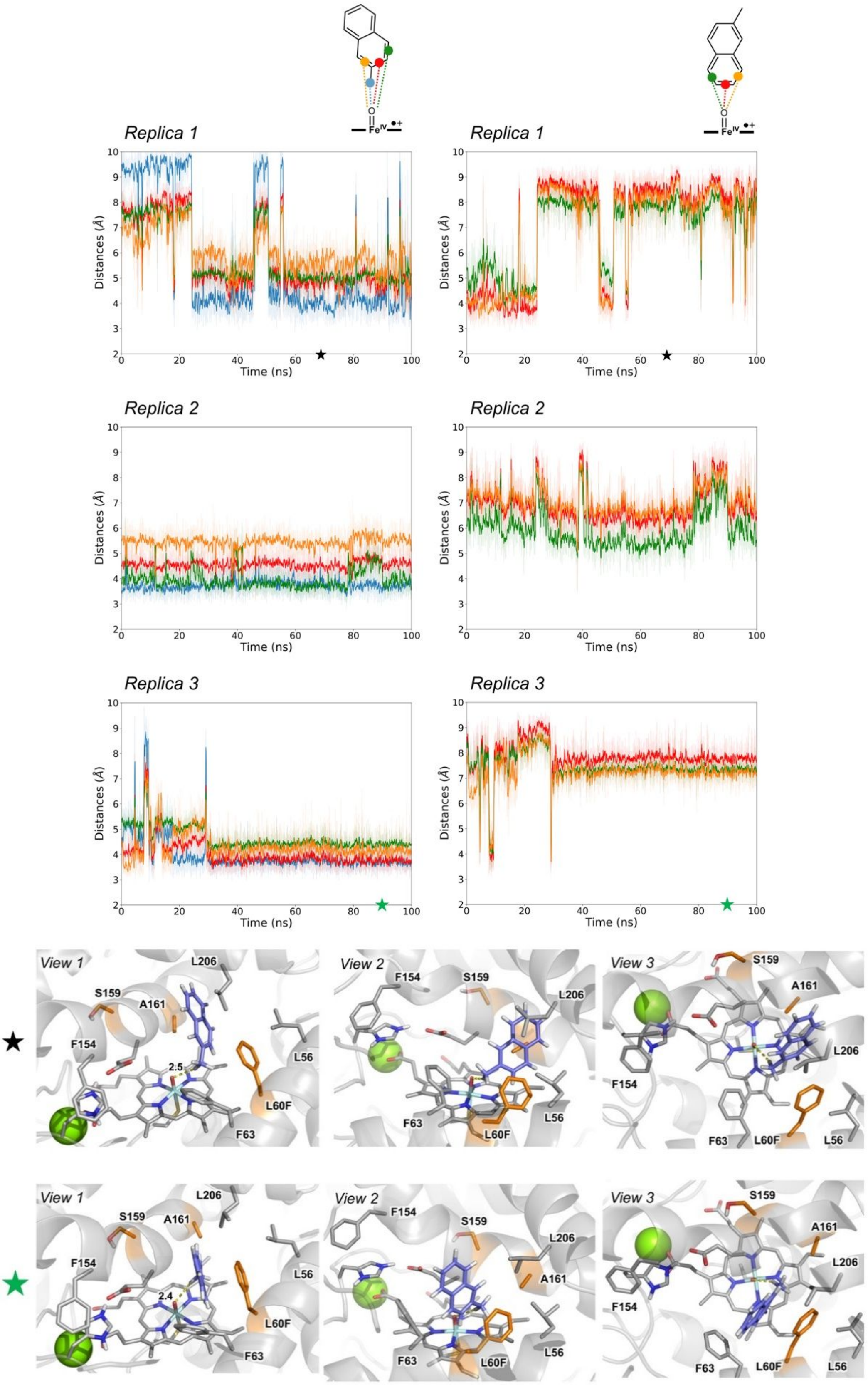

Figure S11 Analysis of 2-methylnaphthalene binding modes through three independent MD replicas in L60F variant. Key distances relevant for hydroxylation and aromatic oxidations are monitored along MD simulations, as described in the schemes. Representative snapshots from MD trajectories (highlighted with a "star" symbol) that describe reactive near attack conformations explored during MDs are shown. Distances and angles are given in angstroms (Å) and degrees (º), respectively. 2-methylnaphthalene bound in L60F variant predominantly explores catalytically relevant binding poses in which the 2-methyl group is placed in a near attack conformation respect to the Fe=O active species. Differences observed for naphthalene derivatives binding modes in different MthUPO variants are discussed in Figure S13.

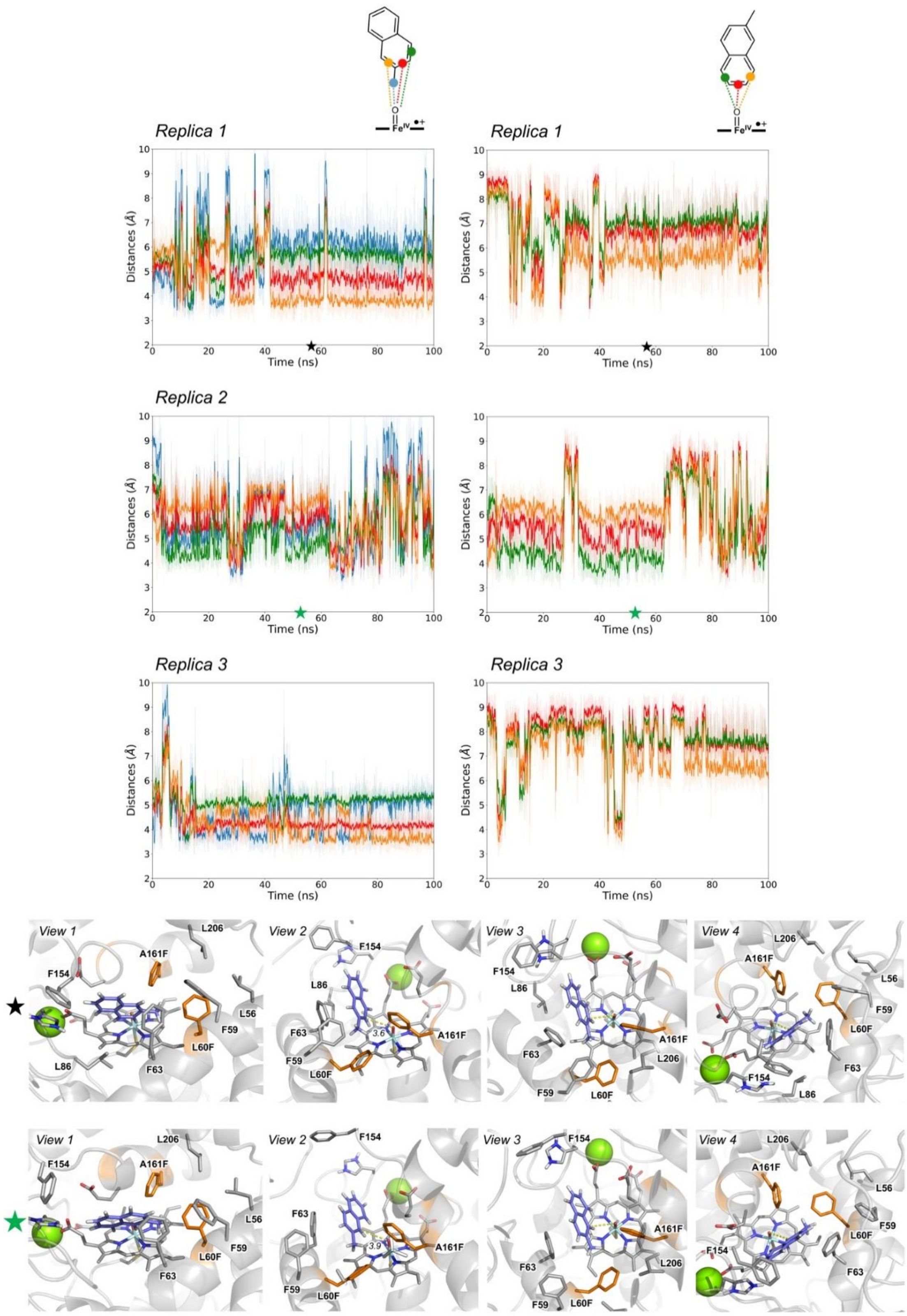

Figure S12 Analysis of 2-methylnaphthalene binding modes through three independent MD replicas in L60F/S159G/A161F variant. Key distances relevant for hydroxylation and aromatic oxidations are monitored along MD simulations, as described in the schemes. Representative snapshots from MD trajectories (highlighted with a "star" symbol) that describe reactive near attack conformations explored during MDs are shown. Distances and angles are given in angstroms (Å) and degrees (º), respectively. 2-methylnaphthalene bound in L60F/S159G/A161F variant predominantly explores catalytically relevant binding poses in which the substituted aromatic ring is placed in a near attack conformation respect to the Fe=O active species. Differences observed for naphthalene derivatives binding modes in different MthUPO variants are discussed in Figure S13.

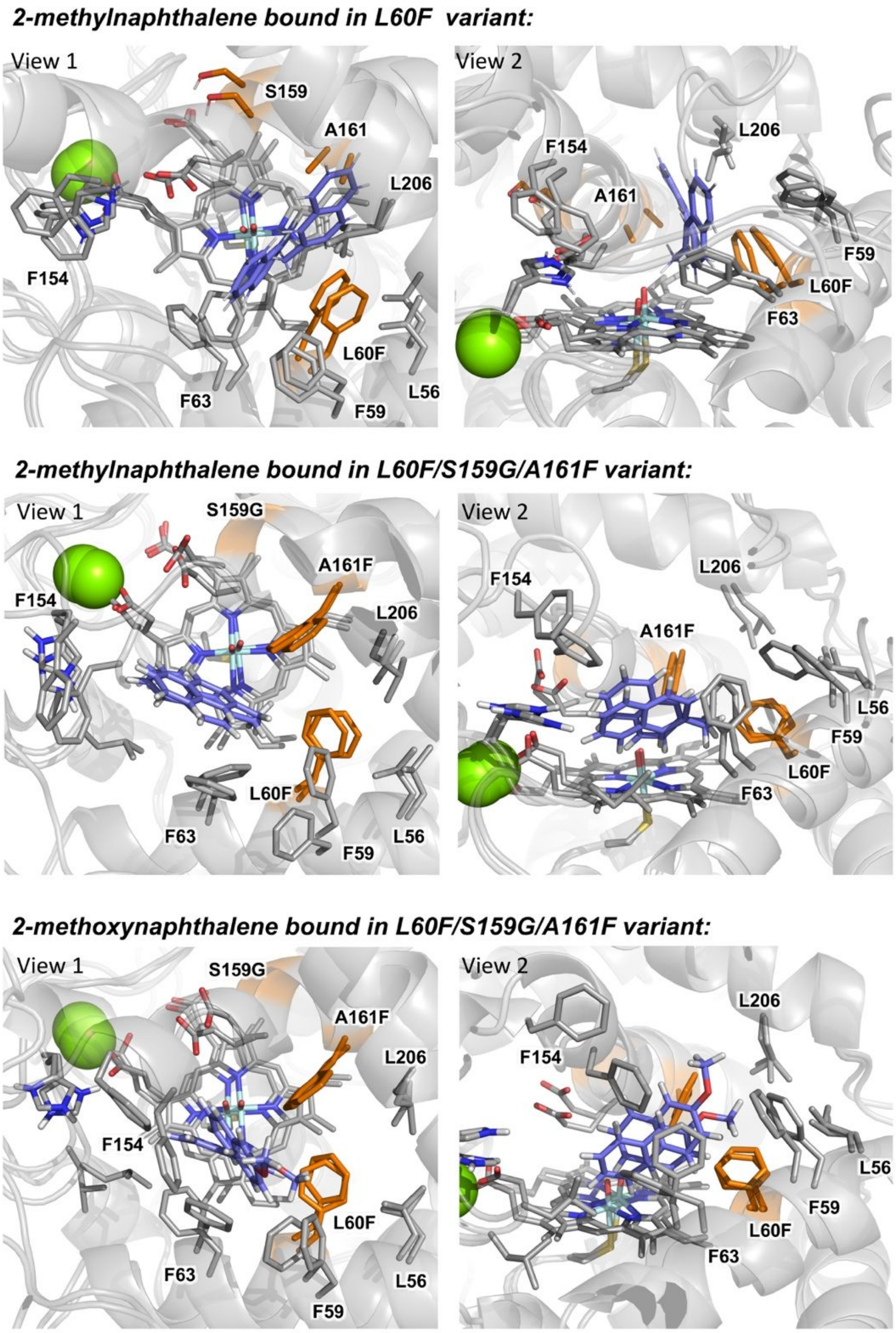

Figure S13 Differences in catalytically relevant binding modes of 2-substituted naphthalene derivatives (2-methlynaphthalene and 2-methoxynaphthalene) in L60F and L60F/S159G/A161F variants as observed from MD simulations (see Figure S11, S12 and S14). Mutated positions are highlighted in orange. 2-methlynaphthalene explores substantially different near attack conformations (NACs) in each variant due to the new introduced mutations. In L60F, 2-methlynaphthalene explores catalytically competent poses in which the 2-methyl group is suitable to directly interacts with Fe=O active species. On the other hand, in L60F/S159G/A161F variant, the substrate is displaced form the former binding position to a new one that resembles the binding mode observed by NBD in this triple mutant (Figure S9). This new binding mode, induced by the presence of bulky A161F mutation, allows the direct interaction between the substituted aromatic ring of 2-methlynaphthalene and the catalytic Fe=O species (see Figure S15). When 2-methoxynaphthalene is bound in L60F/S159G/A161F variant, it occupies the same binding position as 2-methyl derivative. However, because the more bulkier 2-methoxy group, the substrate slightly rotates and preferentially explores catalytically relevant conformations in which the 2-methoxy group is placed far from the haem group and the unsubstituted aromatic ring is placed closer to the Fe=O. Because of this reorientation, the regioselectivity of the oxidation reaction changes from preferential functionalisation at the substituted aromatic ring in 2-methylnaphtalene to the oxidation at the unsubstituted one in 2-methoxynaphthalene when L60F/S159G/A161F variant is used.

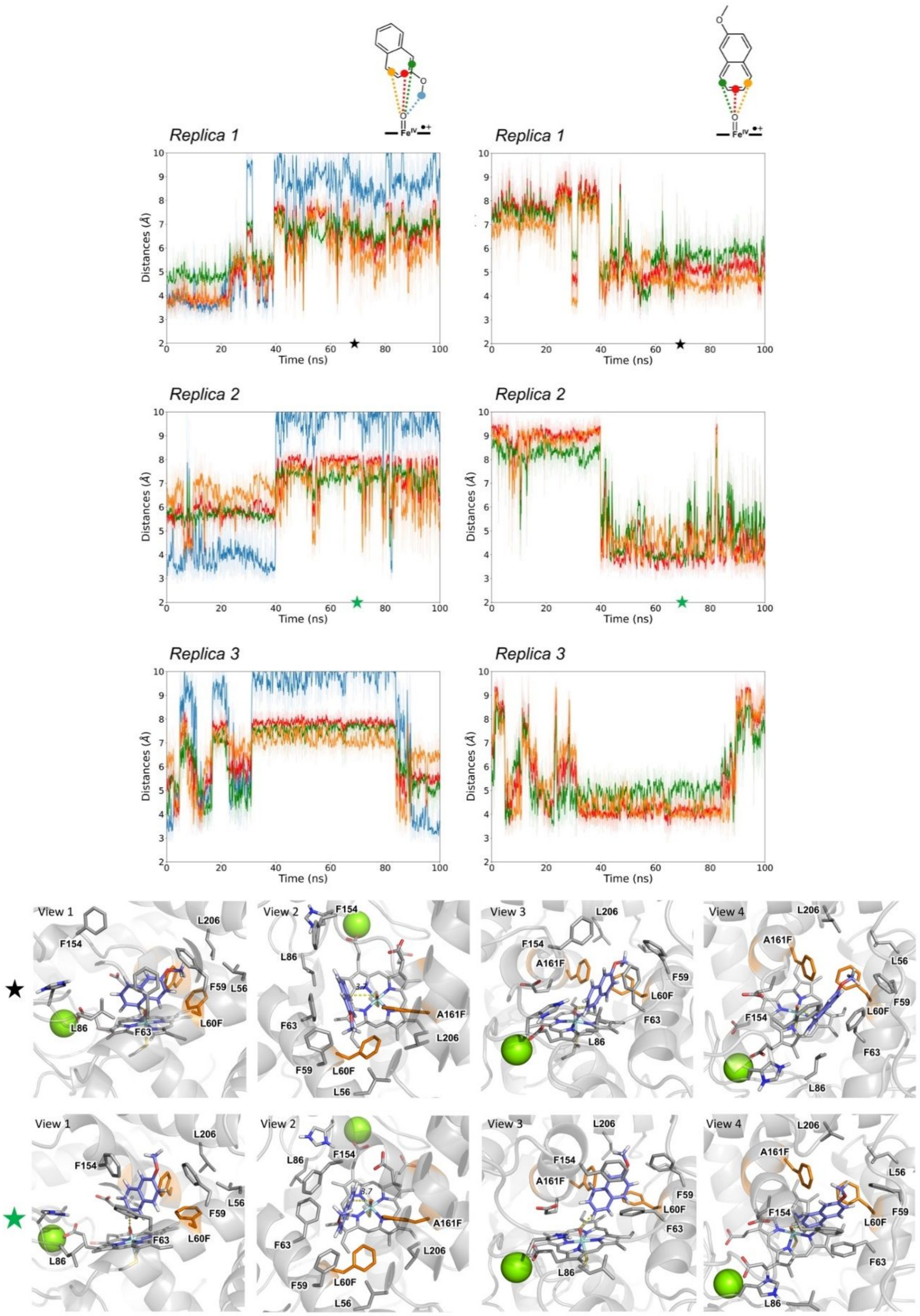

Figure S14. Analysis of 2-methoxynaphthalene binding modes through three independent MD replicas in L60F/S159G/A161F variant. Key distances relevant for hydroxylation and aromatic oxidations are monitored along MD simulations, as described in the schemes. Representative snapshots from MD trajectories (highlighted with a "star" symbol) that describe reactive near attack conformations explored during MDs are shown. Distances and angles are given in angstroms (Å) and degrees (º), respectively.2-methoxynaphthalene bound in L60F/S159G/A161F variant predominantly explores catalytically relevant binding poses in which the unsubstituted aromatic ring is placed in a near attack conformation respect to the Fe=O active species. Differences observed for naphthalene derivatives binding modes in different MthUPO variants are discussed in Figure S13.

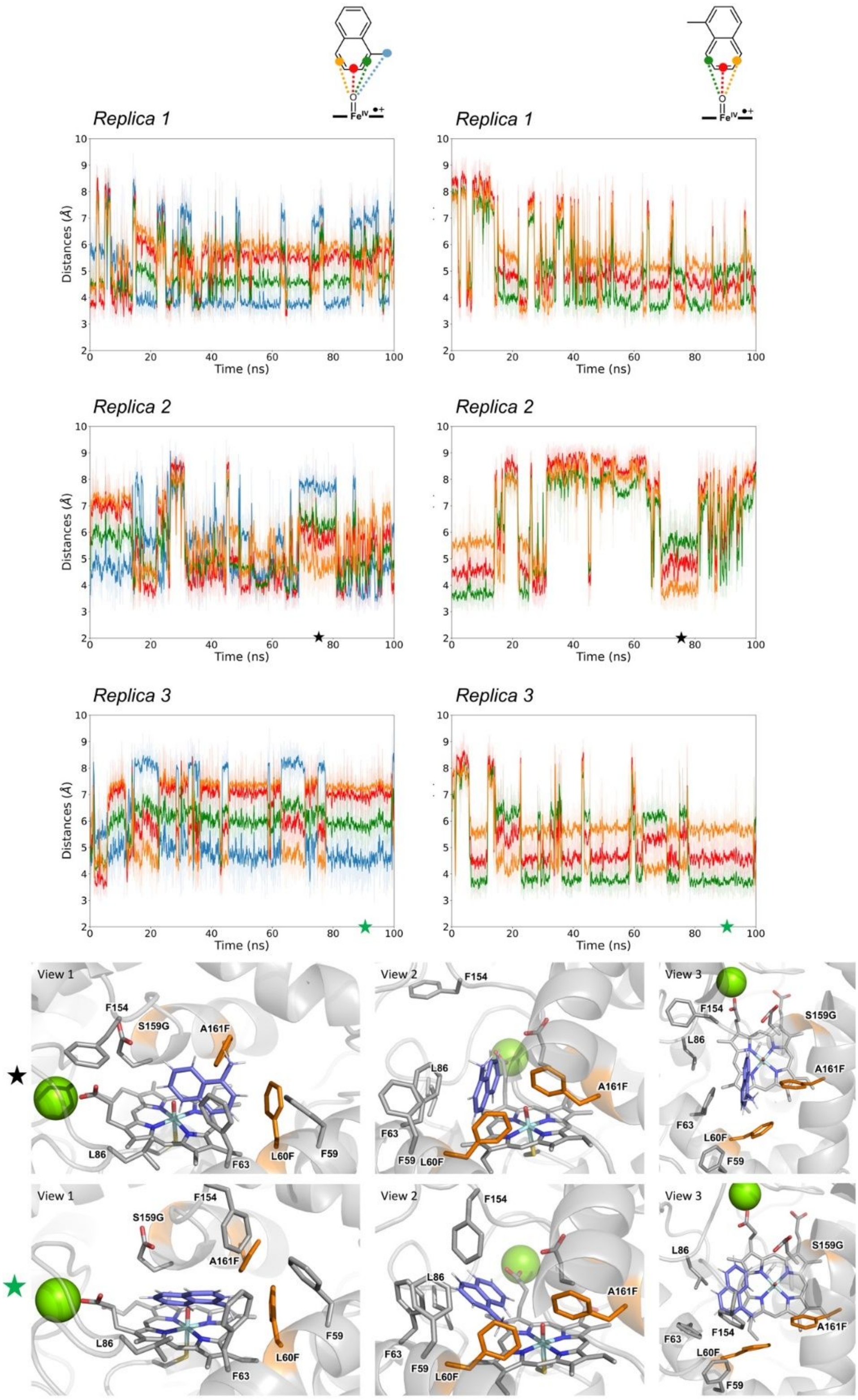

Figure S15. Analysis of 1-methylnaphthalene binding modes through three independent MD replicas in L60F/S159G/A161F variant. Key distances relevant for hydroxylation and aromatic oxidations are monitored along MD simulations, as described in the schemes. Representative snapshots from MD trajectories (highlighted with a "star" symbol) that describe reactive near attack conformations explored during MDs are shown. Distances and angles are given in angstroms (Å) and degrees (º), respectively. 1-methylnaphthalene bound in L60F/S159G/A161F variant predominantly explores catalytically relevant binding poses in which the unsubstituted aromatic ring is placed in a near attack conformation respect to the Fe=O active species.

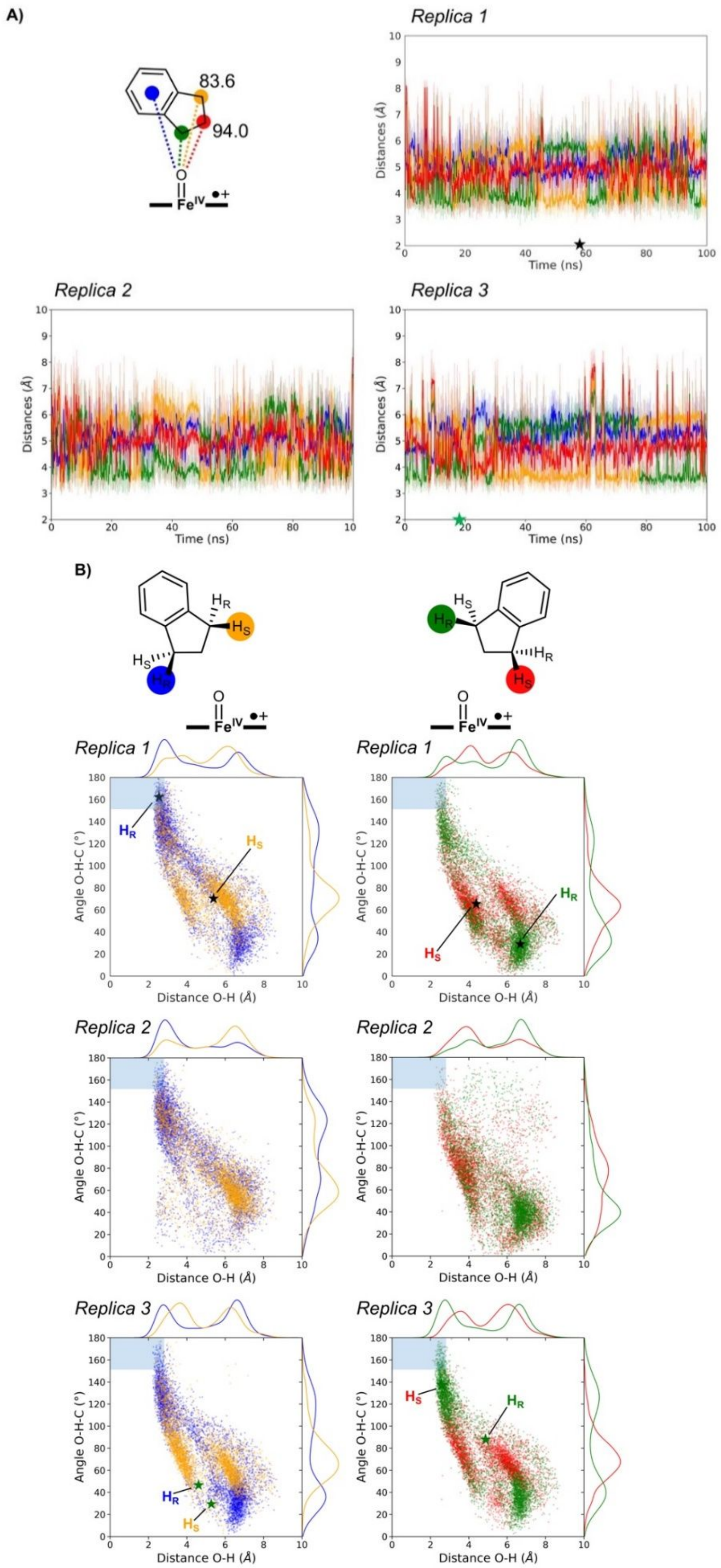
A)
83.6
94.0
Fe^IV •+
Replica 1
Replica 2
Replica 3
Distances (Å)
Time (ns)
B)
H_R
H_S
Fe^IV •+
Replica 1
Replica 2
Replica 3
Angle O-H-C (°)
Distance O-H (Å)

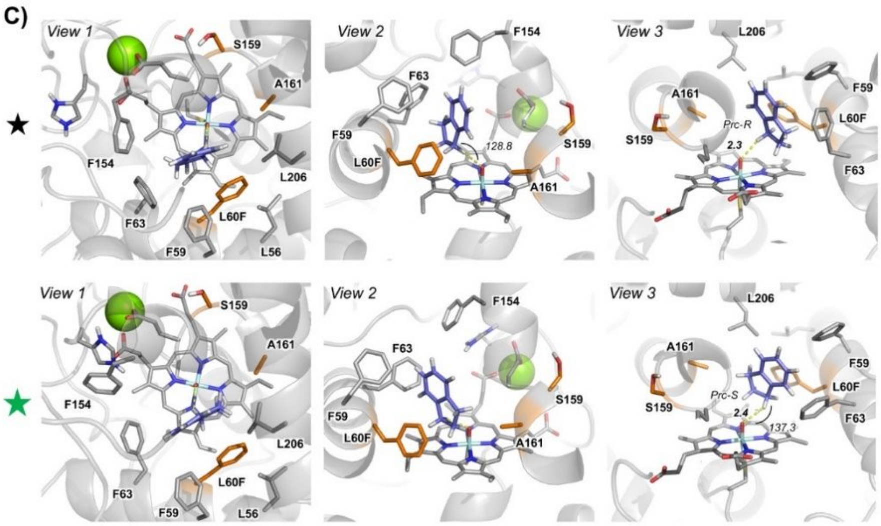

Figure S16. A) Analysis of indane binding modes through three independent MD replicas in L60F variant. Key distances relevant for hydroxylations and aromatic oxidations are monitored along MD simulations, as described in the schemes. Bond Dissociation Energies (BDEs) for C–H bonds at C1 and C2 positions of indane are reported ((U)B3LYP/Def2TZVP/PCM(CH_2Cl_2)// (U)B3LYP/6-31G(d)/PCM(CH_2Cl_2), in kcal·mol^{-1}). **B**) Stereoselectivity analysis of indane oxidation at C1 position from MD simulations. Catalytic distances between O(Fe=O) – H and attack angles (O(Fe=O) – H – C) in the two equivalent *pro*-R and *pro*-S C–H bonds have been monitored, as described in the schemes. **C**) Representative snapshots from MD trajectories (highlighted with an "star" symbol) that describe reactive near attack conformations explored during MDs. Distances and angles are given in angstroms (Å) and degrees (º), respectively. Indane bound in L60F variant predominantly explores catalytically relevant binding poses in which the C1 positions are placed in a near attack conformation respect to the Fe=O active species (**A** and **B**). Calculated C1–H BDEs are significantly lower than those estimated for C2–H, indicating a higher intrinsic reactivity at C1 position. Deeper analysis of the C1 hydroxylation stereopreferences from MD simulations (**B**) demonstrated that *pro-R* C1–H bonds preferentially explore much better near attack conformations (i.e. shorter distances and more optimal attack angles) than *pro-S* C1–H, in line with the high stereoselectivity observed for L60F variant.

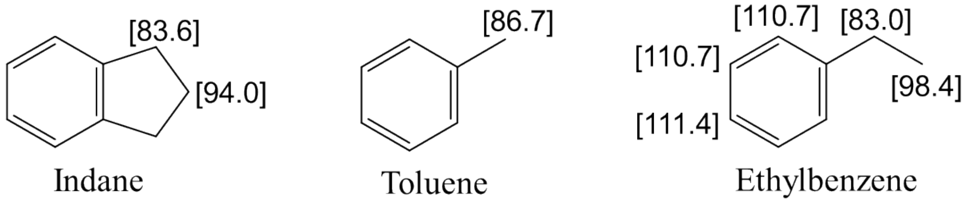

Figure S17. Calculated Bond dissociation Energies (BDEs) for the aliphatic positions of indane, toluene and ethylbenzene, and non-equivalent aromatic positions in ethylbenzene, at the (U)B3LYP-BJD3/Def2TZVP/PCM(dichloromethane) //(U)B3LYP/6-31G(d)/PCM level. BDE values are given in kcal mol^{-1}.

References

(1) Patrick, W. M.; Firth, A. E.; Blackburn, J. M. User-friendly algorithms for estimating completeness and diversity in randomized protein-encoding libraries. *Protein Eng.* **2003**, *16*, 451-457.
